# An *In Vivo* Photo-Cross-Linking Approach Reveals a Homodimerization Domain of Aha1 in *S. cerevisiae*

**DOI:** 10.1371/journal.pone.0089436

**Published:** 2014-03-10

**Authors:** Michael Berg, Annette Michalowski, Silke Palzer, Steffen Rupp, Kai Sohn

**Affiliations:** 1 Fraunhofer IGB, Stuttgart, Germany; 2 IBVT, University of Stuttgart, Stuttgart, Germany; 3 IGVP, University of Stuttgart, Stuttgart, Germany; Berlin Institute of Technology, Germany

## Abstract

Protein-protein interactions play an essential role in almost any biological processes. Therefore, there is a particular need for methods which describe the interactions of a defined target protein in its physiological context. Here we report a method to photo-cross-link interacting proteins in *S. cerevisiae* by using the non-canonical amino acid *p*-azido-L-phenylalanine (pAzpa). Based on the expanded genetic code the photoreactive non-canonical amino acid pAzpa was site-specifically incorporated at eight positions into a domain of Aha1 that was previously described to bind Hsp90 *in vitro* to function as a cochaperone of Hsp90 and activates its ATPase activity. *In vivo* photo-cross-linking to the cognate binding partner of Aha1 was carried out by irradiation of mutant strains with UV light (365 nm) to induce covalent intermolecular bonds. Surprisingly, an interaction between Aha1 and Hsp90 was not detected, although, we could confirm binding of suppressed pAzpa containing Aha1 to Hsp90 by native co-immunoprecipitation. However, a homodimer consisting of two covalently crosslinked Aha1 monomers was identified by mass spectrometry. This homodimer could also be confirmed using *p*-benzoyl-L-phenylalanine, another photoreactive non-canonical amino acid. Crosslinking was highly specific as it was dependent on irradiation using UV light, the exact position of the non-canonical amino acid in the protein sequence as well as on the addition of the non-canonical amino acid to the growth medium. Therefore it seems possible that an interaction of Aha1 with Hsp90 takes place at different positions than previously described *in vitro* highlighting the importance of *in vivo* techniques to study protein-protein interactions. Accordingly, the expanded genetic code can easily be applied to other *S. cerevisiae* proteins to study their interaction under physiological relevant conditions *in vivo*.

## Introduction

To understand the molecular machinery of a cell, methods for the study of protein-protein interactions are essential. The interactome of *S. cerevisiae* was initially explored using the yeast two-hybrid method [1], [2]. Later, high-throughput techniques like tandem-affinity purification combined with mass spectrometry were applied which generated a plethora of data [3]. These studies have contributed greatly to the understanding of the interactome of *S. cerevisiae*. Unfortunately, these methods do not capture the interaction of the protein of interest in the *in vivo* context, the large fusion tag might interfere with the protein function and they are not able to detect transient interactions. Hence, novel methods are needed which offer the opportunity to study protein-protein interactions in the physiological context. In addition, as modifications of the protein of interest like large tags used for tandem affinity purification can lead to changes in structure and function they should be kept as small as possible.

For this purpose the expanded genetic code represents a promising approach to establish novel methods for the analysis of protein-protein interactions. Using the expanded genetic code non-canonical amino acids with novel and unique properties can be incorporated into proteins at defined position *in vivo* using orthogonal suppressor tRNA and amino-acyl tRNA synthetase pairs [4]. Many different fields of application using non-canonical amino acids were already demonstrated like photocleavage of the polypeptide backbone [5], control of protein phosphorylation [6] or modification of a growth hormone with improved clinical performance [7]. To date the expanded genetic code is already established in different organisms including *E. coli* [8], *S. cerevisiae* [9], [10], *P. pastoris* [11], *C. albicans* [12] and mammalian cell lines like Chinese hamster ovary cells [13]. Among dozens of different non-canonical amino acids, there are basically three different types of photoreactive groups, including azido-, diazirin- and benzoyl-groups, which can be incorporated into proteins using the expanded genetic code [14], [15]. For photo-cross-link experiments the non-canonical amino acid pBzpa is widely used to covalently capture the interaction of two proteins [13], [16], [17], [18], [19], [20], [21], [22]. The reason for this is that photolysis of the keto group within the benzophenone can be induced with a relative long wavelength (350–365 nm). It reacts solely with C-H bonds and in case of no reaction this group is able to regenerate [16], [23]. The non-canonical amino acid pAzpa has not yet been widely applied for photo-cross-link experiments *in vivo* [24], [25]. However, pAzpa has the advantage over pBzpa that pAzpa is smaller in size than pBzpa, but can also be activated at 365 nm. Following activation using UV-light a short-lived (∼1 ns) nitrene is formed which is able to form covalent crosslinks with a variety of different chemicals groups. If no interacting partner is available in close vicinity, the group reacts with H_2_O which reduces false positive crosslinks [24], [25], [26].

In the present study we examine the possibility of using the non-canonical amino acid *p*-azido-L-phenylalanine to study protein-protein interactions in *S. cerevisiae*. By now, using the expanded genetic code and pAzpa for photo-cross-linking yet no studies are reported which use a full length protein for protein-protein analysis *in vivo*. For this purpose we chose the well characterized cochaperone Aha1 (activator of Hsp90 ATPase) [27]. Aha1 binds as a monomer to the Hsp90 homodimer. The binding of the Aha1 N-terminal domain to the middle domain of Hsp90 induces a conformational change in Hsp90. This stimulates the ATPase activity of both Hsp90 proteins within the homodimer [28]. The interaction interface of the Aha1 N-terminal domain with the Hsp90 middle domain was characterized by crystallography [29].

Accordingly we specifically incorporated *p*-azido-L-phenylalanine at eight different positions within the binding domain of Aha1 to Hsp90 as suggested by the crystal structure. We confirmed the interaction of the Aha1 variants to Hsp90 by native co-immunoprecipitation. In contrast to the expected Hsp90 and Aha1 heterodimer, a homodimer consisting of two covalently crosslinked Aha1 monomers was identified by mass spectrometry and confirmed by an immunological approach. In addition, this homodimer was also reproduced using the photoreactive amino acid *p*-benzoyl-L-phenylalanine.

## Results

### Site-specific incorporation of pAzpa in to Aha1

**Figure 1:**
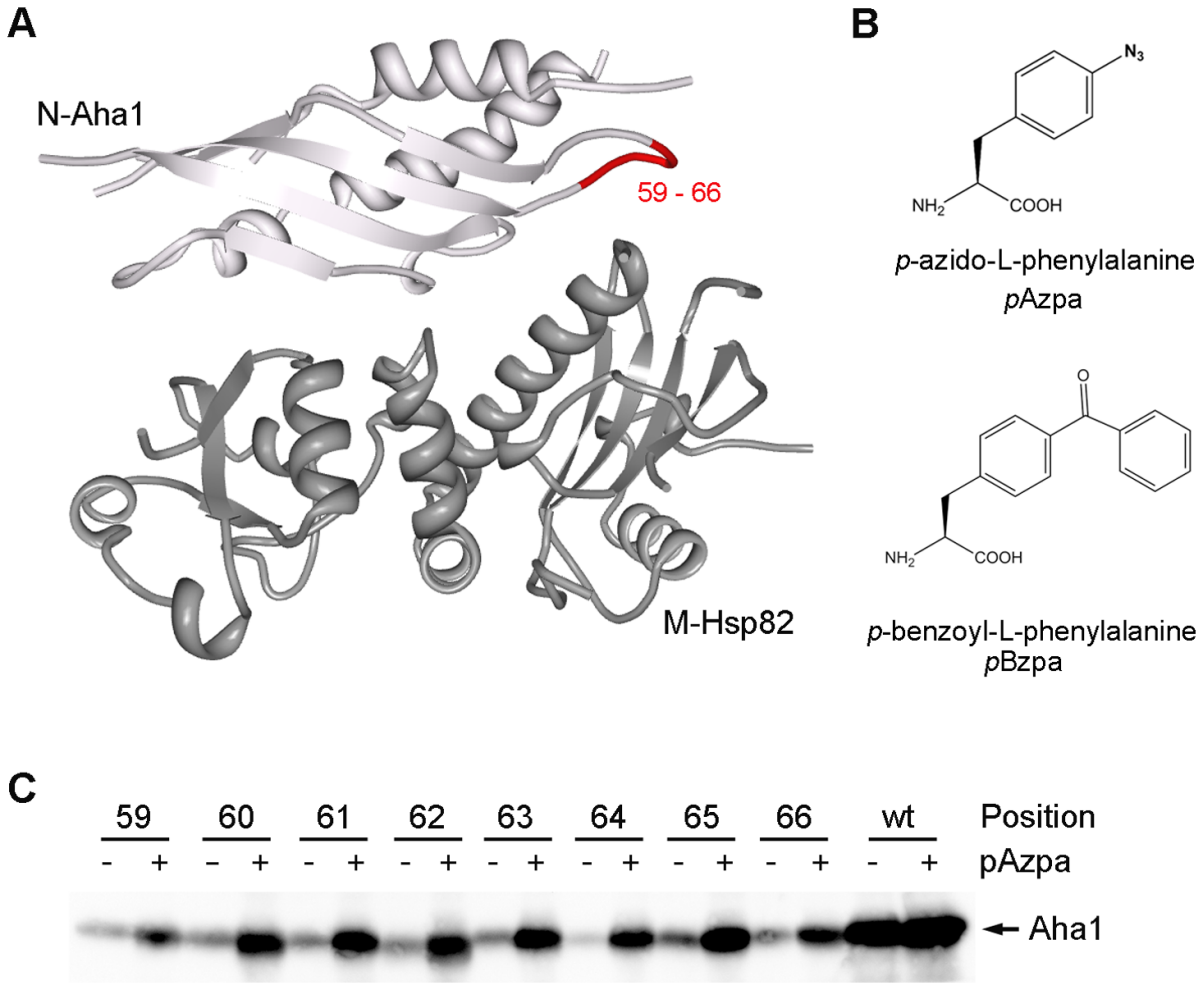
Site-specific incorporation of pAzpa in Aha1. A. Crystal structure of a complex between the middle segment of Hsp90 (M-Hsp90: residues 272–530) and the N-terminal domain of Aha1 (N-Aha1: residues 1–153) deposited at PDB (1USU). The region (59–66) for the incorporation of the non-canonical amino acid within N-Aha1 is highlighted in red. B. Chemical structure of pAzpa and pBzpa. C. Expression of Aha1 in the presence and absence of the non-canonical amino acid pAzpa incorporated at the indicated positions.

For the characterization and application of the expanded genetic code to study protein-protein interactions in *S. cerevisiae* we used the well characterized interaction of Aha1 with Hsp90. Aha1 has been described as a cochaperone that binds to Hsp90 and strongly stimulates its ATPase activity [27], [30]. In *S. cerevisiae* two proteins are expressed Hsp82 and Hsc82 which both belong to the Hsp90 family. They are redundant in function and nearly identical in protein sequence [31]. The interaction between the N-terminal domain of yeast Aha1 (N-Aha1: residues 1–153) and the middle domain of yeast Hsp90 (M-Hsp90: residues 273–530) has been resolved by a crystal structure (Figure 1A) [29]. The hydrophobic side chains of Ile 64, Leu 66 and Phe 100 of Aha1 are suggested to be involved in the core interaction between the N-Aha1 and M-Hsp90. The side chains of the completely conserved motif 59-RKGK-62 among members of the Aha1 family are reported to be orientated towards Hsp90 and stabilize the catalytic loop of Hsp90 in its active conformation [29]. Based on these observations we chose the amino acids 59–66 for substitution with pAzpa to capture an interaction by a covalent crosslink (Figure 1A and B). The Aha1 variants containing an amber codon at the selected positions for suppression were additionally tagged using a V5 epitope and expressed under the control of the *GAL1* promoter from a 2 µ plasmid. The orthogonal pair consisting of an *E. coli* amber suppressor tRNA and *p*-azido-L-phenylalanyl tRNA synthetase (aaRS) necessary for the incorporation of pAzpa were also expressed from a 2 µ plasmid based system [10].

Surprisingly, during our suppression experiments we observed growth deficits of *S. cerevisiae* strain YPH501 in medium containing the non-canonical amino acid pAzpa. Inoculation of the wild type strain YPH501 at a low cell density of OD_600_ = 0.0003 in SC medium containing 2% glucose with various pAzpa concentrations resulted in a significantly inhibited growth rate of cells depending on pAzpa concentration (Figure S1A). This finding indicates an inhibitory effect of the non-canonical amino acid pAzpa. Furthermore, the growth rate of a strain expressing the orthogonal pair (2YA6,64C1) was significantly decreased depending on the pAzpa concentration added to the medium compared to the wild type strain YPH501 (Figure S1B). When pAzpa was replaced by the non-canonical amino acid pBzpa (Figure S1C) the growth rate of the strain was comparable to that of the wild type YPH501. These results indicate that the non-canonical amino acid pAzpa alone and more pronounced in combination with the orthogonal pair leads to adverse effects on growth of *S. cerevisiae* strain YPH501. A similar observation was made for another *S. cerevisiae* strain INVsc1 (data not shown). However, when medium was inoculated with high cell density (OD_600_ = 1) no growth deficits or morphological phenotypes were observed compared with the inoculation described in Figure S1 (data not shown). Accordingly, in the most cases it is sufficient when the expression of the model protein is induced to a specific time point in a dense preculture by adding pAzpa to the medium as described in this study.

Depending on the addition of pAzpa to the medium expression of the eight Aha1 variants containing pAzpa at the defined positions could be clearly detected (Figure 1C). Nevertheless, we observed also some background suppression in the absence of pAzpa. However, depending on the addition of pAzpa to the medium we saw a significantly stronger suppression of the amber stop codon for each Aha1 variant.

To test the availability and reactivity of the azido group incorporated into the Aha1 variant after 24 h of expression two highly chemoselective reactions, the Staudinger ligation and the azide-alkyne cycloaddition (Click Chemistry) [32] were applied for suppressed Aha1 I64X. On the basis of a specific reaction of the azido group with phosphine or alkyne groups various molecules were linked to Aha1 I64X including a triarylphosphine fluorescent dye that was site-specifically coupled to Aha1 I64X by the Staudinger ligation in a crude cell lysate (Figure S2A). In addition, the Cu(I)-catalyzed azide-alkyne cycloaddition was successfully applied for coupling a biotin molecule to immunoprecipitated Aha1 I64X (Figure S2B). These experiments also indicate stability of the azido group to different buffers and environments.

**Figure 2:**
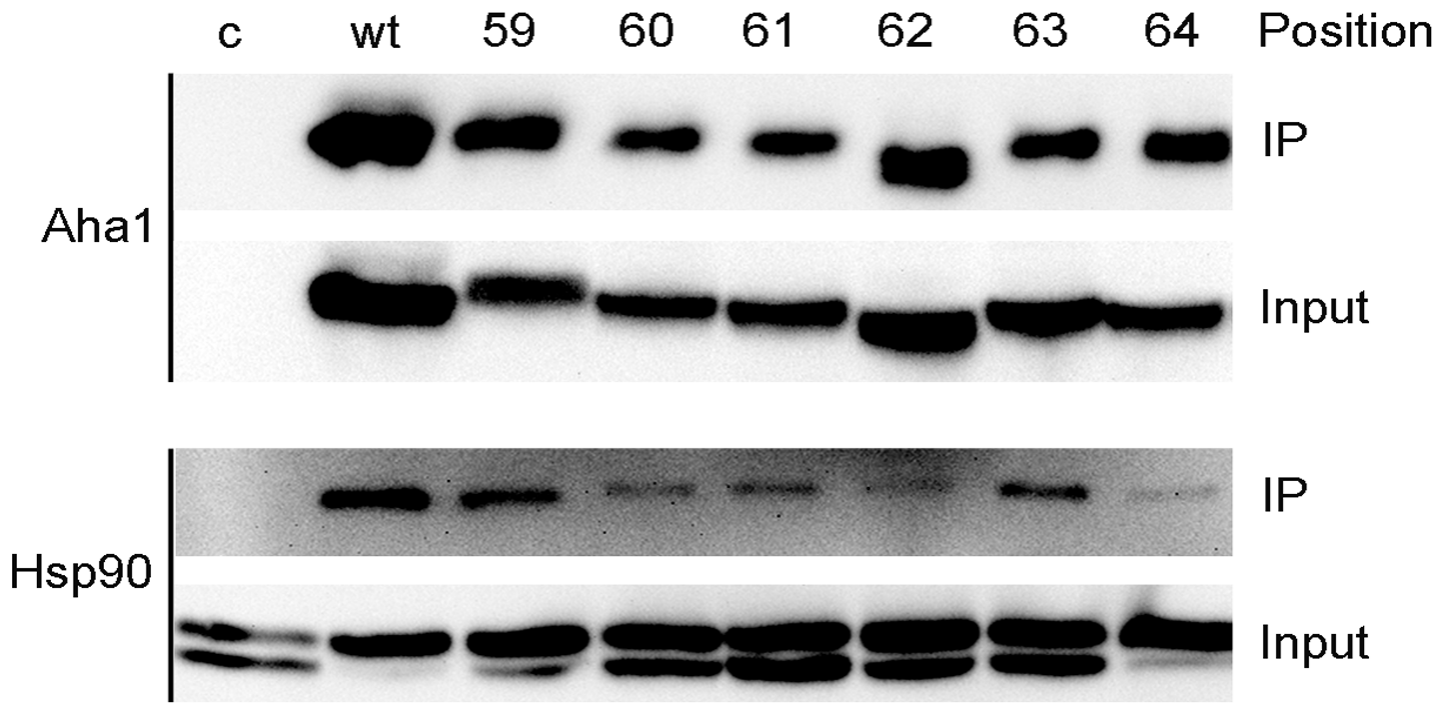
Interaction of pAzpa-containing Aha1 variants with Hsp90. Aha1 variants were expressed in the presence of pAzpa and subsequently immunoprecipitated via their V5-tag. Associated Hsp90 was detected by immunoblotting. Strain 2YA0C1 carrying the empty pYES2/CT vector was used as a control (c). Input = whole protein lysate; IP = eluate after immunoprecipitation.

The substitution of one canonical amino acid by a non-canonical amino acid like pAzpa into the Aha1 protein might influence the three-dimensional (3D)-conformation. This change in conformation might than result in artificial or loss of interactions. To verify that the expressed and modified Aha1 variants were still able to interact with Hsp90, we performed native co-immunoprecipitation. Aha1 variants were precipitated using anti-V5 antibodies and their interaction with Hsp90 was detected by immunoblotting (Figure 2). For all Aha1 variants an interaction with Hsp90 was observed. These results indicate that the replacement of the original canonical amino acids by pAzpa had no negative impact on the interaction between Aha1 variants and Hsp90. When whole protein lysates were separated using 8% polyacrylamide gel two bands were detected with the anti-Hsp90 antibody due to the higher resolution of separation for highly similar Hsp82 and Hsc82 proteins (Figure 2. Hsp90 Input).

### Crosslink formation of Aha1

**Figure 3:**
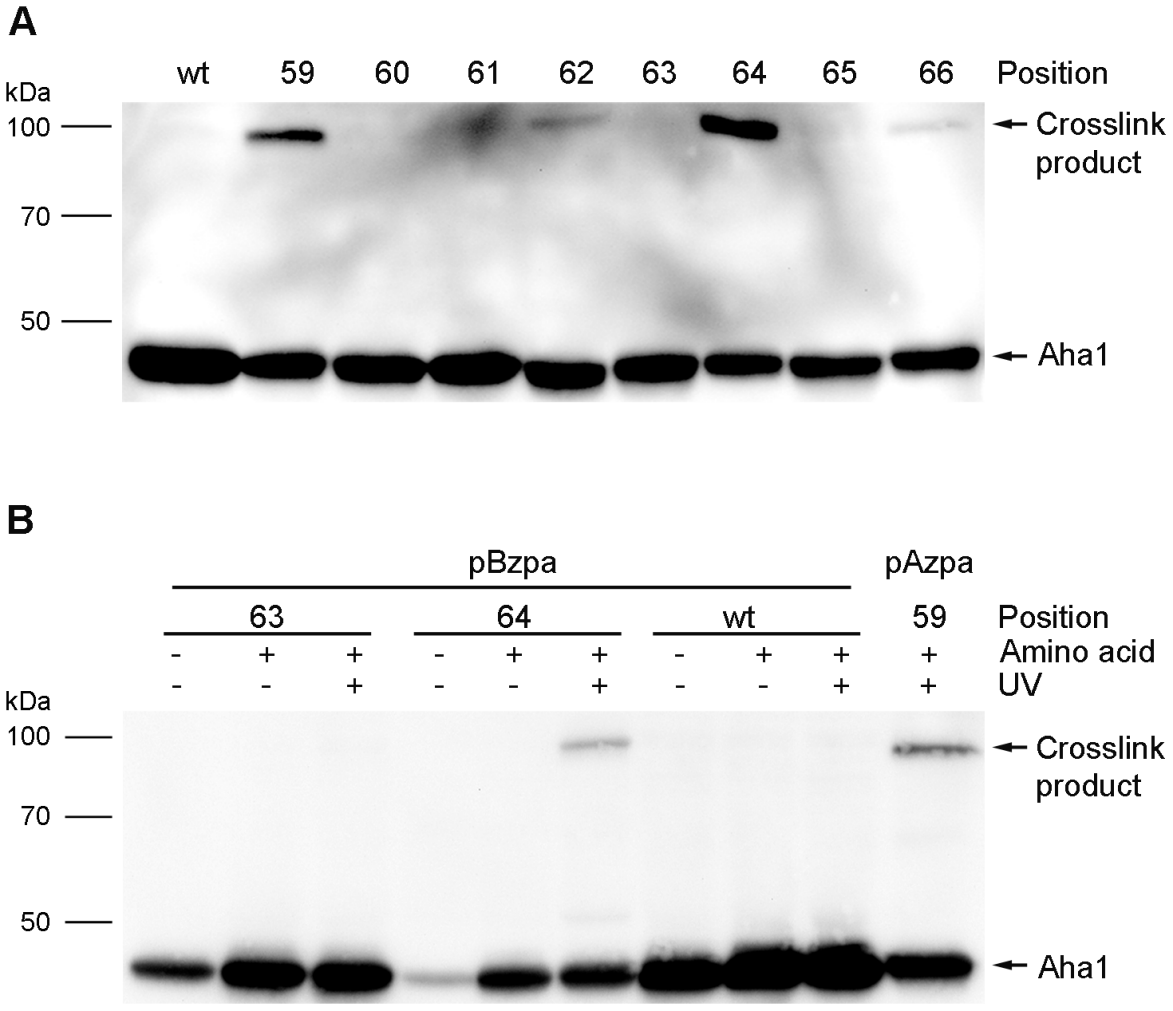
*In vivo* crosslinking of Aha1 variants. A. Following incubation in medium containing pAzpa, cells were exposed to UV light and cell lysates were analyzed by Western blot. The band at 100-canonical amino acid pBzpa was incorporated at position 64. Aha1 variant with pAzpa at position 59 was used as a positive control.

For photo-cross-linking, cells containing the various Aha1 variants and the orthogonal pair were irradiated for 120 min with UV light (365 nm) after 24 hours expression of the Aha1 variants in medium containing galactose and 1 mM pAzpa. Subsequently, proteins were isolated and analyzed by Western blot. The incorporation of pAzpa at the positions 59, 62, 64 and 66 combined with irradiation resulted in the formation of a crosslinked product (Figure 3A). This crosslink product had an apparent molecular weight of approximately 100 kDa and was depending on UV irrediation, the position of substitution within the protein as well as the addition of the non-canonical amino acid pAzpa (Figure S3). No additional crosslinked products could be observed. The amount of crosslink product depends on time of exposure and concentration of pAzpa as reduction of exposure time or lower pAzpa concentration in the medium resulted in decreased crosslink yields (Figure S4). We could not observe spontaneous crosslink product formation for pAzpa without exposure to UV light as reported by Grunbeck [33]. Aryl-azides are usually irradiated with UV light at wavelengths below 310 nm [16], [34]. However, in agreement with Takimoto and co-workers who have shown that the azido group can also be activated at longer wavelengths we photo-cross-linked at 365 nm [25]. Since shorter wavelengths have more energy than longer wavelengths we compared 312 nm and 365 nm wavelength induction with respect to formation of crosslink product after 120 min of irradiation. However we were not able to detect any difference regarding specificity and yield (data not shown).

In addition to pAzpa there are additional photoreactive non-canonical amino acids with photo-crosslink properties which can be incorporated into proteins using the expanded genetic code [4]. Among them is *p*-benzoyl-L-phenylalanine (pBzpa) (Figure 1B). The benzophenone group reacts preferentially with C-H bonds and the excitation is reversible when no covalent bond was formed [23]. In this context, using pBzpa we could successfully confirm the crosslink product observed with pAzpa (Figure 3B). This result shows that the formation of the crosslinked product was independent of the type of photoreactive amino acid used to form a covalent bond between Aha1 I64X and an interacting protein *in vivo*.

For all experiments, mutant Aha1 variants were tagged using a V5/His_6_-tag at the C-terminus. It has already been reported that proteins containing His_6_-tags may differ from their wild-type counterparts in dimerization properties [35], therefore we checked whether crosslink product formation is depending on the His_6_-tag. In this context we expressed the wild-type Aha1, Aha1 R59X, Aha1 V63X and Aha1 I64X either with the V5-, HA- or Flag-tag using the same expression vector and linker sequence between the Aha1 and the respective tag. Using these constructs, crosslink experiments revealed that the specific type of C-terminal tag had no influence on the formation of the observed crosslink product (Figure S5). For Aha1 R59X and Aha1 I64X the crosslink product could be reproduced independent of the tag, whereas no crosslink product formation was observed for wild-type Aha1 and Aha1 V63X that were used as negative control.

### Identification of the Aha1 interacting partner

**Figure 4:**
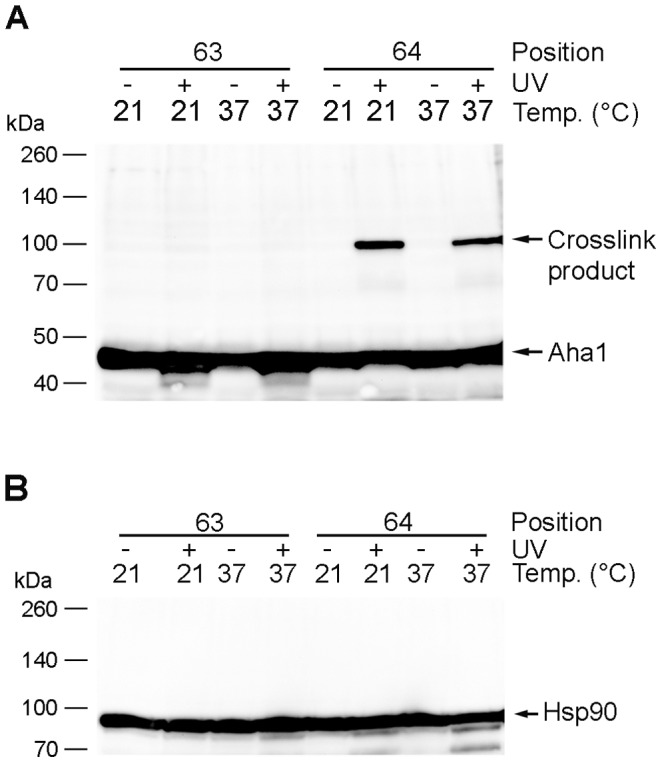
Crosslink product formation is not dependent on temperature. A. Successful crosslink product formation could be observed both at 21 and 37 degree. No other crosslinks could be detected using an anti-V5 antibody. B. Identical samples as in A were analyzed using anti-Hsp90 antibody. No additional crosslink product band above the Hsp90 signal was detectable.

The interaction of Aha1 with Hsp90 has already been demonstrated with various methods like yeast two-hybrid assay or co-immunoprecipitation [27], [36]. Moreover, this interaction is also structurally well characterized by crystal structures [28], [29]. Based on such a crystal structure we expected to capture Hsp90 by a covalent crosslink through the incorporation of pAzpa at one of the positions 59–66 in Aha1. Although the apparent molecular weight of crosslinked proteins are difficult to predict, this heterodimer should have a size of approximately 120 kDa which can be deduced from the individual molecular masses of Hsp82 (81 kDa) and Aha1 (39 kDa). Instead, we found that the crosslink product has an apparent molecular mass of 100 kDa. To test whether Hsp90 was the interaction partner of Aha1 we used Western blot analysis using anti-Hsp90 antibodies to examine whether Hsp90 is part of the crosslink product. Surprisingly, it was not possible to detect any crosslink product with two different anti-Hsp90 antibodies although the Hsp90 monomer gave a clear and strong signal (Figure 4B). Since Hsp90 was first described as a heat shock protein [31] and the expression of both proteins increase under stress conditions like elevated temperatures [37], we also cultivated Aha1 V63X and Aha1I64X strains at 21°C in galactose medium for 24 hours followed by a heat shock at 37°C for 20 min for a fraction of the culture. Afterwards, both 21°C and 37°C cultures were exposed to UV light (365 nm). Under these conditions no band in addition to the Aha1 monomer and the already observed crosslink product could be detected using an anti-V5 antibody (Figure 4A). Again, analysis with an anti-Hsp90 antibody solely showed the signal for the Hsp90 monomer, but no crosslink product at 100 kDa (Figure 4B). These experiments indicate that the crosslinked dimer does not depend on temperature and that Hsp90 could not be found covalently associated to Aha1 variants.

**Figure 5:**
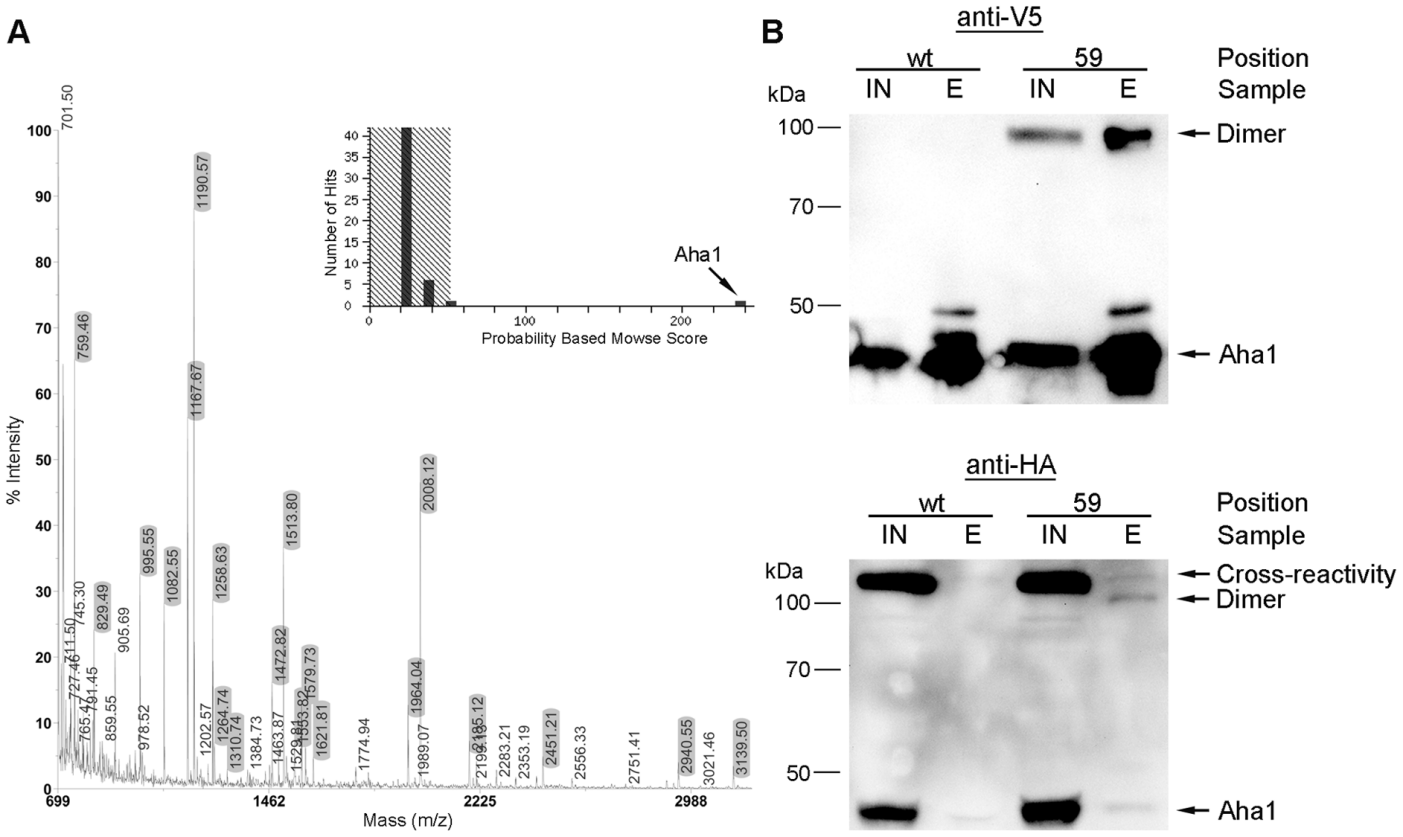
Identification of the Aha1 cross-linked interaction partner. A. Tryptic fragmentation of the crosslinked product band that was cut out from SDS-PAGE, revealed peptides only matching to Aha1. Mass peaks that could be unambiguously assigned to Aha1 protein are highlighted in grey. No additional proteins could be detected using MASCOT's “Probability Based MOWSE Score”, indicating that the crosslink product represents a homodimer consisting of two Aha1 proteins. B. Endogenous Aha1 was fused to a HA-tag at its C-terminus and simultaneously expressed with the pAzpa-containing Aha1 mutant (59) or wild-type Aha1 (wt) fused to a V5-tag. Following irradiation with UV light Aha1-V5 was immunoprecipitated using an anti-V5 antibody. The corresponding eluates (E) were analyzed using anti-V5 and anti-HA antibodies. A crosslink product generated by the Aha1 mutant with pAzpa at the position 59 could be detected by both antibodies.

As our immunological data demonstrate that Hsp90 most likely is not the interacting partner in the covalently crosslinked protein dimer, we therefore immunoprecipitated in a next step the crosslink product in sufficient yields for analysis by mass spectrometry (Figure 5A). Following separation by SDS-PAGE and in gel digest of the cut out band of the crosslink product we found that almost all dominant peaks except for 905.69 could clearly be assigned to Aha1 (highlighted in grey), with about 81% of the Aha1 protein sequence including the C-terminal tag was covered (Figure S6). No further protein in the sample in addition to Aha1 with a significant Probability Based Mowse Score could be detected. As another covalently bound protein had to be present in the sample in stoichiometric amounts, the fact that no other protein could be detected strongly suggests a homodimerization of Aha1 with itself. The assumption that a homodimer was covalently crosslinked is supported by the apparent molecular weight which is twice as big as of a single Aha1 monomer (regarding the apparent migration behavior in SDS-PAGE). In good agreement to a putative homodimer we could also demonstrate by two-dimensional (2D) gel electrophoresis, that the homodimer shows exactly the same isoelectric points compared to the monomer (Figure S7). To confirm this homodimer we also tagged an endogenously expressed Aha1 with a C-terminal HA tag. Cells simultaneously expressing the constitutively expressed Aha1-HA as well as galactose-induced Aha1 R59X containing a V5 epitope were exposed to UV light. The corresponding V5-immunoprecipitated dimer could be detected by Western blot using the anti-HA and anti-V5 antibody (Figure 5B). No dimer was detected upon immunoprecipitation with wild type V5-tagged Aha1. This result confirms the crosslink product as a homodimer consisting of two covalently crosslinked Aha1 monomers. In additional experiments we also found that crosslink product formation was not due to aggregation of misfolded Aha1 proteins within the cell as most of the crosslinked product was found in the supernatant of high speed centrifugation representing the soluble fraction (Figure S8A). Crosslink formation also was unlikely to be co-translationally generated at poly-ribosomes as inhibition of translation using cycloheximide for different periods of times prior to UV-irradiation still results in the formation of the specific crosslink product (Figure S8B).

## Discussion

The study of protein-protein interactions by using position-specific, incorporated photo-cross-linkers such as the two non-canonical amino *p*-azido-L-phenylalanine or *p*-benzoyl-L-phenylalanine also holds numerous advantages over unspecific chemical compounds such as formaldehyde. By position-specific incorporation of non-canonical amino acids, each region of the protein can be specifically examined for interactions, including those interactions positioned in the interior of the proteins and for this reason are not accessible for chemical agents. In addition, the rapid reaction kinetic after induction with UV light permits the capture of transient and weak interactions, thereby minimizing false-positive results. Importantly, the replacement of only one amino acid is the smallest modification possible in a protein.

For this work we applied the expanded genetic code to study protein-protein interactions in *S. cerevisiae* using full length proteins. In this context the non-canonical amino acid pAzpa was incorporated at defined positions in the cochaperone Aha1. Aha1 was selected as a model protein because of its well characterized and proven interaction to Hsp90 by yeast two-hybrid [27], [38], co-immunoprecipitation [27], [36], multidimensional NMR technique [28] and crystallisation [29]. Depending on addition of pAzpa to the medium, we were able to incorporate the non-canonical amino acid at each of the selected positions. In contrast, recently Nehring et al. showed that they were not able to suppress an amber stop codon with pAzpa in *S. cerevisiae* [39]. This finding is very surprising as by using the same orthogonal pair as Nehring et al. that has been initially introduced by the group of Schultz we have expressed different proteins having incorporated the non-canonical amino acid pAzpa in *S. cerevisiae* strain YPH501. In addition, we also used the commercially available strain INVsc1 mentioned in this study in experiments to successfully express full length Gal4 with pAzpa at different positions. We cannot explain the lack of incorporation of pAzpa into proteins in *S. cerevisiae* found by Nehring et al., but would like to point out that the concept of an expanded genetic code to incorporate pAzpa and pBzpa into proteins in *S. cerevisiae in vivo* in fact is feasible.

Prior to carrying out photo-cross-link experiments we first confirmed the preservation of the interaction between the Aha1 variants and Hsp90 by native co-immunoprecipitation. This control was crucial as non-canonical amino acid or a fused tag may change the interaction behavior of a protein. Interestingly, we were able to generate a covalent crosslink including the positions described to be involved in the core interaction between Aha1 and Hsp90 (64 and 66) [29]. We were surprised to observe a crosslinked Aha1 homodimer as we clearly expected to find a heterodimer consisting of Aha1 and Hsp90, because the interaction interface was previously well characterized by a crystal structure [29] and confirmed by a multidimensional NMR technique [28]. However, we do not at all rule out an interaction of Aha1 to Hsp90 as we still find this interaction by native co-immunoprecipitation. It seems reasonable to speculate that an additional interface might be also probable for the interaction of both proteins under these experimental conditions *in vivo*. In general, the fact that *in vivo* photo-cross-link results do not necessarily agree with *in vitro* findings is in line with findings of other groups [40], for example. In this study, we have successfully demonstrated the applicability of the non-canonical amino acid *p*-azido-L-phenylalanine in *S. cerevisiae* to investigate protein-protein interactions in their natural context using the expanded genetic code. We found that an Aha1 domain which has been described to be implicated in Hsp90 binding *in vitro* was involved in homo-dimerization *in vivo*. These results therefore highlight the need for *in vivo* techniques for protein interaction analysis.

## Materials and Methods

### Strains and Materials

*S. cerevisiae* strain YPH501 (MATa/α *ura3–52*/*ura3–52 lys2–801^amber^*/l*ys2–801^amber^ ade2–101^ochre^*/*ade2–101^ochre^ trp1–Δ63*/*trp1–Δ63 his3–Δ200*/*his3–Δ200 leu2–Δ1*/*leu2–Δ1*; Agilent Technologies) and *E. coli* strain DH5α (F-, *endA1*, *hsdR17* (rk-, mk-), *supE44*, *thi-1*, *recA1*, *gyrA96*, *relA1*, Δ(*argF-lac*)*U169*, *λ-*, *φ80dlacZΔM15*) were used in this study. The non-canonical amino acids pAzpa and pBzpa were purchased from Bachem (Weil am Rhein, Germany). Unless otherwise indicated chemicals were from Roth (Karlsruhe, Germany) or Sigma-Aldrich (Taufkirchen, Germany). All enzymes were purchased from New England Biolabs (Frankfurt/Main, Germany). The monoclonal mouse anti-V5 antibody, the monoclonal mouse anti-Hsp90 antibody and the polyclonal rabbit anti-HA antibody were purchased from Acris (Herford, Germany). Horseradish peroxidase-conjugated sheep anti-mouse IgG and goat anti-rabbit IgG was from GE Healthcare (Freiburg, Germany). Primers used in this study are listed in Table S1.

### Plasmid construction and site-directed mutagenesis

The endogenous *AHA1* sequence was amplified using primers AHA1_BamHI and AHA1_XbaI containing cleavage sites for *Bam*HI or *Xba*I at their 5′-ends. The PCR fragment was digested simultaneously with *Bam*HI and *Xba*I subsequently cloned into the *Bam*HI and *Xba*I sites of the expression vector pYES2/CT (Invitrogen). The wild-type *AHA1* gene in the vector was mutagenized using the Phusion site-directed mutagenesis protocol (Thermo Scientific), to generate a series of clones with an amber stop codon at positions 59–66 (Table S1). The correct sequences of the corresponding plasmids (Table S3) were verified by sequencing.

### *S. cerevisiae* transformation

Transformation was carried out according to standard LiAc/SS carrier DNA/PEG protocols [41]. Vectors encoding the different *AHA1* variants were co-transformed with the vector encoding the aminoacyl tRNA synthetase/tRNA pair (pAR3-PGK1+3SUP4-tRNA or pBR2-PGK1+3SUP4-tRNA) into *S. cerevisiae* strain YPH501 to create strains for photo-crosslink-experiments (Table S2). Positive clones were selected on synthetic complete medium lacking uracil and tryptophan (SC -URA - TRP; 2% glucose, 0.17% yeast nitrogen base w/o amino acid and w/o ammonium sulfate (Difco Laboratories), 0.5% ammonium sulfate, 0.01% each adenine, arginine, cysteine, leuine, lysine, threonine and 0.005% each aspartic acid, histidine, isoleucine, methionine, phenylalanine, proline, serine, tyrosine, valine).

### PCR-based tagging

PCR-based tagging was carried out according to the method of Janke with several modifications [42]. The Primers S2_AHA1 and S3_AHA1 with flanking regions of homology were used to amplify the cassette from vector pYM45 for C terminal tagging (Table S1). The amplification was performed in 50 µl reaction with 1× Phusion HF buffer, 0.2 mM dNTP's, 0.5 µM each primer, 1 U of Phusion HF DNA polymerase and approximately 100 ng cassette vector. PCR was performed with an initial denaturation at 98°C (30 sec), followed by 12 cycles of 98°C (10 sec), 60°C (30 sec), 72°C (65 sec) and 18 cycles of 98°C (10 sec), 72°C (65 sec). Following gel purification (QIAquick Gel Extraction Kit) the amplicon was transformed into *S. cerevisiae* strain YPH501. Positive clones were selected on YPD-plates containing 200 µg/ml geneticin (G418).

### *In vivo* photo-cross-linking and cell lysis

Individual colonies of Aha1 variants were grown at 30°C in SC medium containing 2% glucose, but lacking uracil and tryptophan up to an OD_600_ of approximately 2. For the expression of Aha1 cells were reinoculated to an OD_600_ of 1 into SC medium containing 2% galactose and 1 mM pAzpa (dissolved to 1 M in 1 M NaOH) or pBzpa (dissolved to 0.2 M in 1 M NaOH), but lacking uracil and tryptophan and were grown for 24 hours at 30°C. Afterwards cultures were irradiated for 2 h by 365 nm UV light (Bio-Link BLX 365 Crosslinker, 5×8 W bulbs). Cells from a 20 ml culture were pelleted by centrifugation. The pellet volume was estimated and equal volume of glass beads (0.25–0.5 mm) and lysis buffer (50 mM Tris-Cl pH 7.5, 200 mM NaCl, 5 mM EDTA, 1% (v/v) Triton X-100) were added to the pellet. Cells were lysed by three 5 min cycles of vortexing in the cold room. Foam was centrifuged at 16,060 g for 2 min between the vortex steps. Lysates were cleared by centrifugation for 10 min at 16.060 g and 4°C.

### Separation of soluble from insoluble proteins and cycloheximide-chase assay

Aha1 I64X (2YA6,64C1) was expressed and crosslink products were formed by irradiation with UV light. Disruption was carried out using a Mixer Mill MM 200 (Retsch) with a shaking frequency of 30 s^−1^. The resulting cell powder was resuspended in aqueous buffer without detergent (50 mM Tris-Cl pH 7.5, 500 mM NaCl, 1 mM PMSF, 1× protease inhibitor cocktail without EDTA (Roche)). Soluble proteins were separated from the insoluble proteins by a 100,000× g centrifugation for 30 minutes. Insoluble proteins were solved in an insoluble protein buffer (50 mM Tris-Cl pH 7.5, 8 M urea, 2% SDS, 150 mM NaCl, 2 mM DTT, 1 mM PMSF, 1× protease inhibitor cocktail without EDTA (Roche)).

For translational inhibition assays using cycloheximide Aha1 I64X (2YA6,64C1) was expressed and cycloheximide (final concentration 50 µg/ml) was added to the culture prior to UV-irradiation for different periods of time.

### Western blot analysis

For Western blot analysis proteins were precipitated from cell lysates according to Wessel and Flugge [43]. Protein concentration was determined by the Dc Protein Assay (Bio-Rad). Per sample 75 µg total protein was separated by 6% or 8% SDS-PAGE [44]. After electrophoresis, the proteins were blotted onto a PVDF membrane (Immobilon P with a pore size of 0.45 µm, Millipore). The proteins were probed with either monoclonal mouse anti-V5 antibody (1∶5,000), polyclonal rabbit anti-HA antibody (1∶5,000) or monoclonal mouse anti-Hsp90 antibody (1∶1,000). Detection was carried out using peroxidase coupled sheep anti-mouse antibody (1∶5,000) or peroxidase coupled goat anti-rabbit antibody (1∶5,000) and the ECL Plus Western Blotting Substrate (Pierce) using the LAS-1000 CCD camera (Fuji Photo Film).

### Immunoprecipitation

After 24 h of Aha1 expression under the corresponding conditions, cells were harvested by centrifugation and immediately frozen in liquid nitrogen. Disruption was carried out using a Mixer Mill MM 200 (Retsch) with a shaking frequency of 30 s^−1^. The resulting cell powder was resuspended in 1 ml lysis buffer (20 mM Tris-Cl pH 7.5, 1× protease inhibitor cocktail without EDTA (Roche)). Cell debris was removed by centrifugation at 16,060 g for 7 min at 4°C. For each immunoprecipitation 500 µl of the supernatant was used according to the V5 tagged Protein Purification Kit protocol (MBL International Corporation). Four washing steps were performed with wash buffer (20 mM Tris-Cl pH 7.5, 1× protease inhibitor cocktail without EDTA (Roche), 100 mM NaCl, 5 mM MgCl_2_, 0.1% (v/v) Tween 20). Two specific elution steps were carried out using the V5 peptide.

### Mass spectrometry

In order to achieve a sufficient amount of crosslink product for MS analysis, strain 2YA6,63C1 and 2YA6,64C1 were cultured in 200 ml SC-TRP-URA medium in the presence of pAzpa as already described. The cultures were split after UV irradiation in ten 20 ml fractions and cells were lysed corresponding to the already described glass bead protocol. For each immunoprecipitation 500 µl of the supernatant was used according to the V5 tagged Protein Purification Kit protocol (MBL International Corporation). The elution of the crosslink product was performed with 50 µl 4× Laemmli buffer (200 mM Tris-Cl pH 8.0, 5% (w/v) SDS, 40% (v/v) glycerol, 20% (v/v) β-mercaptoethanol, tip of bromophenol blue). The first two 50 µl eluates were used to elute the remaining eight samples. Afterwards the two 50 µl eluates, each represent five immunoprecipitations, were pooled and separated in a large format 6% SDS-PAGE (PROTEAN® II xi Cell from Bio-Rad). The gels were stained using the FOCUS-FAST Silver Kit (G-Biosciences) which is compatible to MS analysis. The corresponding crosslink product band was cut out, proteins were digested and identified by MALDI-TOF/MS according to standard protocols (TopLab (Martinsried, Germany)) [45].

## Supporting Information

Figure S1 Effects of non-canonical amino acids and the orthogonal pair on cell growth. All experiments were performed in SC medium containing glucose as a carbon source. For all experiments two controls were used without addition of NaOH (control) and the addition of the required amount of 1 M NaOH to solubilize pAzpa (dashed line). A. Growth curves of the wild-type strain YPH501 in the presence of different pAzpa concentrations. B. Growth curves of strain 2YA6,64C1 transfected with plasmids for mutant Aha1 I64X and the orthogonal pair for site-specific incorporation of pAzpa in the presence of different pAzpa concentrations. C. Growth of strain 2YA6,64C1 in medium containing different concentrations of pBzpa.
(TIF)

Figure S2 Site-specific conjugation of chemical compounds using the azido-group. A. Chemoselective ligation of the triarylphosphine fluorescent dye using the Staudinger ligation reaction. Aha1 I64X (2YA6,64C1) was expressed in the presence of pAzpa, afterwards cells were disrupted and the ligation reaction was performed using whole cell lysate. Labeled Aha1 protein was immunoprecipitated with anti-V5 antibody. The immunoprecipitate (lane 6) and 50 µg (lane 3) or 100 µg (lane 4) of lysate protein were separated by the SDS-PAGE. Fluorescence was read out by using a fluorescence scanner. Wild-type Aha1 (2YA6C1) was subjected to the same labeling reaction and was used as a negative control (50 µg protein lysate = lane 1; 100 µg protein lysate = lane 2; immunoprecipitated eluate = lane 5). B. Chemoselective conjugation of a biotin molecule using the azide-alkyne cycloaddition (Click Chemistry). Aha1 I64X (2YA6,64C1) was expressed in the presence of pAzpa, afterwards cells were disrupted and Aha1 proteins were immunoprecipitated with the anti-V5 antibody. The eluate was then used for the biotin labeling procedure. Samples before (lane 3) and after (lane 4) labeling were analyzed by Western blot using streptavidin-HRP, showing that biotin was successfully linked to Aha1 at position 64. Labeled Aha1 proteins were also detected with the anti-V5 antibody (lane 6). The wild-type Aha1 (2YA6C1) was used as the negative control before (lane 1) and after (lane 2) labeling; lane 5 represents the same sample as lane 2 but analyzed using anti-V5 antibodies.
(TIF)

Figure S3 Crosslink product formation depends on position, UV irradiation and the presence of the non-canonical amino acid pAzpa. Wild-type Aha1 (2YA6C1) was used as a negative control and expression of Act1 was used as loading control. Detection of the Aha1 variants was carried out with a monoclonal mouse anti-V5 antibody and the detection of Act1 using a monoclonal mouse anti-Act1 antibody. A. Positions showing crosslink product formation. B. All positions with no crosslink product formation.
(TIF)

Figure S4 Time of UV irradiation and pAzpa concentration have an influence on crosslink formation. A. Strain 2YA6,64C1 was exposed for different periods of time to UV light. Significant crosslink product formation could be observed after 15 min of irradiation with UV light. Irradiation up to 120 min leads to an increased yield of the formation of the crosslinked product. B. Cells were cultivated in medium with different pAzpa concentrations. The yield of crosslink product formation correlates to the concentration of pAzpa given to the medium. No crosslink product formation was observed without pAzpa.
(TIF)

Figure S5 The C-terminal tag has no effect on crosslink product formation. Independent of the C-terminal tag, crosslink product formation could be confirmed with Aha1 variant 59 and 64. A. Different Aha1 variants were tagged with the V5 epitope at the C-terminus. Aha1 I64X (2YA6,64C1) C-terminally tagged with V5 epitope and poly-histidine tag was used as control. B. Aha1 variants C-terminally tagged with the HA epitope detected with anti-HA antibodies. C. Aha1 variants C-terminally tagged with the FLAG epitope and detected with anti-FLAG antibodies.
(TIF)

Figure S6 A. Sequence coverage of the Aha1 I64X protein. Protein regions which could be covered by matching peptides are highlighted in red. Position 64 is marked in green. B. Peak table showing all found masses matching to the Aha1 I64X protein sequence.
(TIF)

Figure S7 Analysis of the Aha1 homodimer by 2-D electrophoresis. Aha1 I64X (2YA6,64C1) was immunoprecipitated before (left) and after irradiation (right blot) by UV light. Eluates were separated by 2-D electrophoresis and analyzed by Western blot with monoclonal mouse anti-V5 antibody. The crosslink product migrates to the same isoelectric points as the Aha1 monomer.
(TIF)

Figure S8 Crosslink products are found in the soluble fraction and are not formed co-translationally at poly-ribosomes. A. Separation of soluble from insoluble proteins was performed to show that the crosslink product is not formed due to the aggregation of misfolded Aha1 proteins within the cell. Aha1 I64X (2YA6,64C1) was expressed, crosslink products were formed by irradiation with UV light and soluble proteins were separated from the insoluble proteins by a 100,000× g centrifugation. Different volumes of each fraction were analyzed by Western Blot using mouse monoclonal anti-V5 antibody. Crosslink product as well as monomer was mainly found in the soluble fraction. B. Cycloheximide-chase assay were performed to demonstrate that crosslinking does not occur co-translationally. At different time points following addition of cycloheximide cells were exposed to UV light. Crosslink products could be formed to each time point after inhibition of the translation in comparable yields.
(TIF)

Table S1 Primer sequences for cloning and mutagenesis of AHA1.
(DOCX)

Table S2 *S. cerevisiae* strains generated in this study.
(DOCX)

Table S3 Plasmids used for this work.
(DOCX)

## References

[1] Uetz P, Giot L, Cagney G, Mansfield TA, Judson RS, et al (2000) A comprehensive analysis of protein-protein interactions in Saccharomyces cerevisiae. Nature 403: 623–627. 10688190 10.1038/35001009

[2] Ito T, Chiba T, Ozawa R, Yoshida M, Hattori M, et al (2001) A comprehensive two-hybrid analysis to explore the yeast protein interactome. Proc Natl Acad Sci U S A 98: 4569–4574. 11283351 10.1073/pnas.061034498 PMC31875

[3] Gavin AC, Bosche M, Krause R, Grandi P, Marzioch M, et al (2002) Functional organization of the yeast proteome by systematic analysis of protein complexes. Nature 415: 141–147. 11805826 10.1038/415141a

[4] Liu CC, Schultz PG (2010) Adding New Chemistries to the Genetic Code. Annu Rev Biochem 10.1146/annurev.biochem.052308.105824 20307192

[5] Peters FB, Brock A, Wang J, Schultz PG (2009) Photocleavage of the polypeptide backbone by 2-nitrophenylalanine. Chem Biol 16: 148–152. 19246005 10.1016/j.chembiol.2009.01.013 PMC2714363

[6] Lemke EA, Summerer D, Geierstanger BH, Brittain SM, Schultz PG (2007) Control of protein phosphorylation with a genetically encoded photocaged amino acid. Nat Chem Biol 3: 769–772. 17965709 10.1038/nchembio.2007.44

[7] Cho H, Daniel T, Buechler YJ, Litzinger DC, Maio Z, et al (2011) Optimized clinical performance of growth hormone with an expanded genetic code. Proc Natl Acad Sci U S A 108: 9060–9065. 21576502 10.1073/pnas.1100387108 PMC3107295

[8] Wang L, Brock A, Herberich B, Schultz PG (2001) Expanding the genetic code of Escherichia coli. Science 292: 498–500. 11313494 10.1126/science.1060077

[9] Chin JW, Cropp TA, Anderson JC, Mukherji M, Zhang Z, et al (2003) An expanded eukaryotic genetic code. Science 301: 964–967. 12920298 10.1126/science.1084772

[10] Chen S, Schultz PG, Brock A (2007) An improved system for the generation and analysis of mutant proteins containing unnatural amino acids in Saccharomyces cerevisiae. J Mol Biol 371: 112–122. 17560600 10.1016/j.jmb.2007.05.017

[11] Young TS, Ahmad I, Brock A, Schultz PG (2009) Expanding the genetic repertoire of the methylotrophic yeast Pichia pastoris. Biochemistry 48: 2643–2653. 19265424 10.1021/bi802178k

[12] Palzer S, Bantel Y, Kazenwadel F, Berg M, Rupp S, et al (2013) An Expanded Genetic Code in Candida albicans to study Protein-Protein Interactions in vivo. Eukaryot Cell 10.1128/EC.00075-13 PMC3675983 23543672

[13] Hino N, Okazaki Y, Kobayashi T, Hayashi A, Sakamoto K, et al (2005) Protein photo-cross-linking in mammalian cells by site-specific incorporation of a photoreactive amino acid. Nat Methods 2: 201–206. 15782189 10.1038/nmeth739

[14] Xie J, Schultz PG (2006) A chemical toolkit for proteins–an expanded genetic code. Nat Rev Mol Cell Biol 7: 775–782. 16926858 10.1038/nrm2005

[15] Dennehey BK, Noone S, Liu WH, Smith L, Churchill ME, et al (2013) The C terminus of the histone chaperone Asf1 cross-links to histone H3 in yeast and promotes interaction with histones H3 and H4. Mol Cell Biol 33: 605–621. 23184661 10.1128/MCB.01053-12 PMC3666882

[16] Chin JW, Martin AB, King DS, Wang L, Schultz PG (2002) Addition of a photocrosslinking amino acid to the genetic code of Escherichia coli. Proc Natl Acad Sci U S A 99: 11020–11024. 12154230 10.1073/pnas.172226299 PMC123203

[17] Mori H, Ito K (2006) Different modes of SecY-SecA interactions revealed by site-directed in vivo photo-cross-linking. Proc Natl Acad Sci U S A 103: 16159–16164. 17060619 10.1073/pnas.0606390103 PMC1621050

[18] Das S, Oliver DB (2011) Mapping of the SecA.SecY and SecA.SecG interfaces by site-directed in vivo photocross-linking. J Biol Chem 286: 12371–12380. 21317284 10.1074/jbc.M110.182931 PMC3069440

[19] Forne I, Ludwigsen J, Imhof A, Becker PB, Mueller-Planitz F (2012) Probing the conformation of the ISWI ATPase domain with genetically encoded photoreactive crosslinkers and mass spectrometry. Mol Cell Proteomics 11: M111 012088. 10.1074/mcp.M111.012088 PMC3322568 22167269

[20] Majmudar CY, Lee LW, Lancia JK, Nwokoye A, Wang Q, et al (2009) Impact of nonnatural amino acid mutagenesis on the in vivo function and binding modes of a transcriptional activator. J Am Chem Soc 131: 14240–14242. 19764747 10.1021/ja904378z PMC4182099

[21] Chen HT, Warfield L, Hahn S (2007) The positions of TFIIF and TFIIE in the RNA polymerase II transcription preinitiation complex. Nat Struct Mol Biol 14: 696–703. 17632521 10.1038/nsmb1272 PMC2483787

[22] Krishnamurthy M, Dugan A, Nwokoye A, Fung YH, Lancia JK, et al (2011) Caught in the act: covalent cross-linking captures activator-coactivator interactions in vivo. ACS Chem Biol 6: 1321–1326. 21977905 10.1021/cb200308e PMC3245988

[23] Wittelsberger A, Mierke DF, Rosenblatt M (2008) Mapping ligand-receptor interfaces: approaching the resolution limit of benzophenone-based photoaffinity scanning. Chem Biol Drug Des 71: 380–383. 18312550 10.1111/j.1747-0285.2008.00646.x PMC2570705

[24] Chin JW, Santoro SW, Martin AB, King DS, Wang L, et al (2002) Addition of p-azido-L-phenylalanine to the genetic code of Escherichia coli. J Am Chem Soc 124: 9026–9027. 12148987 10.1021/ja027007w

[25] Takimoto JK, Adams KL, Xiang Z, Wang L (2009) Improving orthogonal tRNA-synthetase recognition for efficient unnatural amino acid incorporation and application in mammalian cells. Mol Biosyst 5: 931–934. 19668857 10.1039/b904228h

[26] Seok WK, Meyer TJ (2004) Stepwise oxidation of anilines by cis-[RuIV(bpy)2(py)(O)]2+. Inorg Chem 43: 5205–5215. 15310196 10.1021/ic0302985

[27] Panaretou B, Siligardi G, Meyer P, Maloney A, Sullivan JK, et al (2002) Activation of the ATPase activity of hsp90 by the stress-regulated cochaperone aha1. Mol Cell 10: 1307–1318. 12504007 10.1016/s1097-2765(02)00785-2

[28] Retzlaff M, Hagn F, Mitschke L, Hessling M, Gugel F, et al (2010) Asymmetric activation of the hsp90 dimer by its cochaperone aha1. Mol Cell 37: 344–354. 20159554 10.1016/j.molcel.2010.01.006

[29] Meyer P, Prodromou C, Liao C, Hu B, Roe SM, et al (2004) Structural basis for recruitment of the ATPase activator Aha1 to the Hsp90 chaperone machinery. Embo J 23: 1402–1410. 15039704 10.1038/sj.emboj.7600141 PMC381413

[30] Wandinger SK, Richter K, Buchner J (2008) The Hsp90 chaperone machinery. J Biol Chem 283: 18473–18477. 18442971 10.1074/jbc.R800007200

[31] Borkovich KA, Farrelly FW, Finkelstein DB, Taulien J, Lindquist S (1989) hsp82 is an essential protein that is required in higher concentrations for growth of cells at higher temperatures. Mol Cell Biol 9: 3919–3930. 2674684 10.1128/mcb.9.9.3919-3930.1989 PMC362454

[32] Agard NJ, Baskin JM, Prescher JA, Lo A, Bertozzi CR (2006) A comparative study of bioorthogonal reactions with azides. ACS Chem Biol 1: 644–648. 17175580 10.1021/cb6003228

[33] Grunbeck A, Huber T, Sachdev P, Sakmar TP (2011) Mapping the ligand-binding site on a G protein-coupled receptor (GPCR) using genetically encoded photocrosslinkers. Biochemistry 50: 3411–3413. 21417335 10.1021/bi200214r PMC3099303

[34] Dorman G, Prestwich GD (2000) Using photolabile ligands in drug discovery and development. Trends Biotechnol 18: 64–77. 10652511 10.1016/s0167-7799(99)01402-x

[35] Wu J, Filutowicz M (1999) Hexahistidine (His6)-tag dependent protein dimerization: a cautionary tale. Acta Biochim Pol 46: 591–599. 10698267

[36] Mollapour M, Tsutsumi S, Donnelly AC, Beebe K, Tokita MJ, et al (2010) Swe1Wee1-dependent tyrosine phosphorylation of Hsp90 regulates distinct facets of chaperone function. Mol Cell 37: 333–343. 20159553 10.1016/j.molcel.2010.01.005 PMC2824606

[37] Gasch AP, Spellman PT, Kao CM, Carmel-Harel O, Eisen MB, et al (2000) Genomic expression programs in the response of yeast cells to environmental changes. Mol Biol Cell 11: 4241–4257. 11102521 10.1091/mbc.11.12.4241 PMC15070

[38] Millson SH, Truman AW, King V, Prodromou C, Pearl LH, et al (2005) A two-hybrid screen of the yeast proteome for Hsp90 interactors uncovers a novel Hsp90 chaperone requirement in the activity of a stress-activated mitogen-activated protein kinase, Slt2p (Mpk1p). Eukaryot Cell 4: 849–860. 15879519 10.1128/EC.4.5.849-860.2005 PMC1140089

[39] Nehring S, Budisa N, Wiltschi B (2012) Performance analysis of orthogonal pairs designed for an expanded eukaryotic genetic code. PLoS One 7: e31992. 22493661 10.1371/journal.pone.0031992 PMC3320878

[40] Pham ND, Parker RB, Kohler JJ (2012) Photocrosslinking approaches to interactome mapping. Curr Opin Chem Biol 10.1016/j.cbpa.2012.10.034 PMC3594551 23149092

[41] Gietz RD, Woods RA (2006) Yeast transformation by the LiAc/SS Carrier DNA/PEG method. Methods Mol Biol 313: 107–120. 16118429 10.1385/1-59259-958-3:107

[42] Janke C, Magiera MM, Rathfelder N, Taxis C, Reber S, et al (2004) A versatile toolbox for PCR-based tagging of yeast genes: new fluorescent proteins, more markers and promoter substitution cassettes. Yeast 21: 947–962. 15334558 10.1002/yea.1142

[43] Wessel D, Flugge UI (1984) A method for the quantitative recovery of protein in dilute solution in the presence of detergents and lipids. Anal Biochem 138: 141–143. 6731838 10.1016/0003-2697(84)90782-6

[44] Laemmli UK (1970) Cleavage of structural proteins during the assembly of the head of bacteriophage T4. Nature 227: 680–685. 5432063 10.1038/227680a0

[45] Shevchenko A, Wilm M, Vorm O, Mann M (1996) Mass spectrometric sequencing of proteins silver-stained polyacrylamide gels. Anal Chem 68: 850–858. 8779443 10.1021/ac950914h

