# Supplementary figures and images for: An In Vivo Photo-Cross-Linking Approach Reveals a Homodimerization Domain of Aha1 in S. cerevisiae
Source: PLoS One. 2014 Mar 10;9(3):e89436. doi: 10.1371/journal.pone.0089436 (PMC3948627; doi:10.1371/journal.pone.0089436)

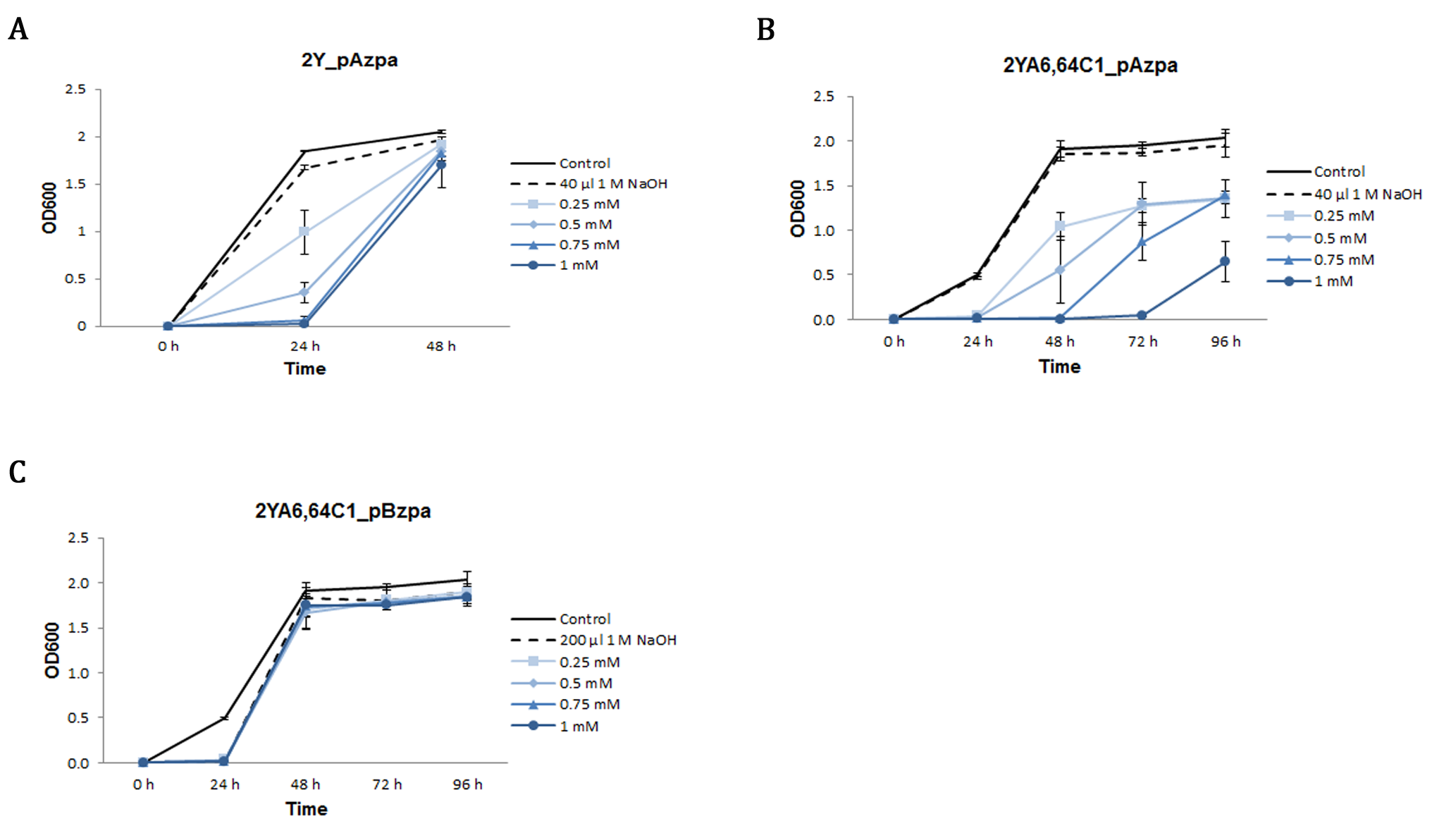

Supplement: Figure S1 — Effects of non-canonical amino acids and the orthogonal pair on cell growth. All experiments were performed in SC medium containing glucose as a carbon source. For all experiments two controls were used without addition of NaOH (control) and the addition of the required amount of 1 M NaOH to solubilize pAzpa (dashed line). A. Growth curves of the wild-type strain YPH501 in the presence of different pAzpa concentrations. B. Growth curves of strain 2YA6,64C1 transfected with plasmids for mutant Aha1 I64X and the orthogonal pair for site-specific incorporation of pAzpa in the presence of different pAzpa concentrations. C. Growth of strain 2YA6,64C1 in medium containing different concentrations of pBzpa. (TIF) [file pone.0089436.s001.tif]

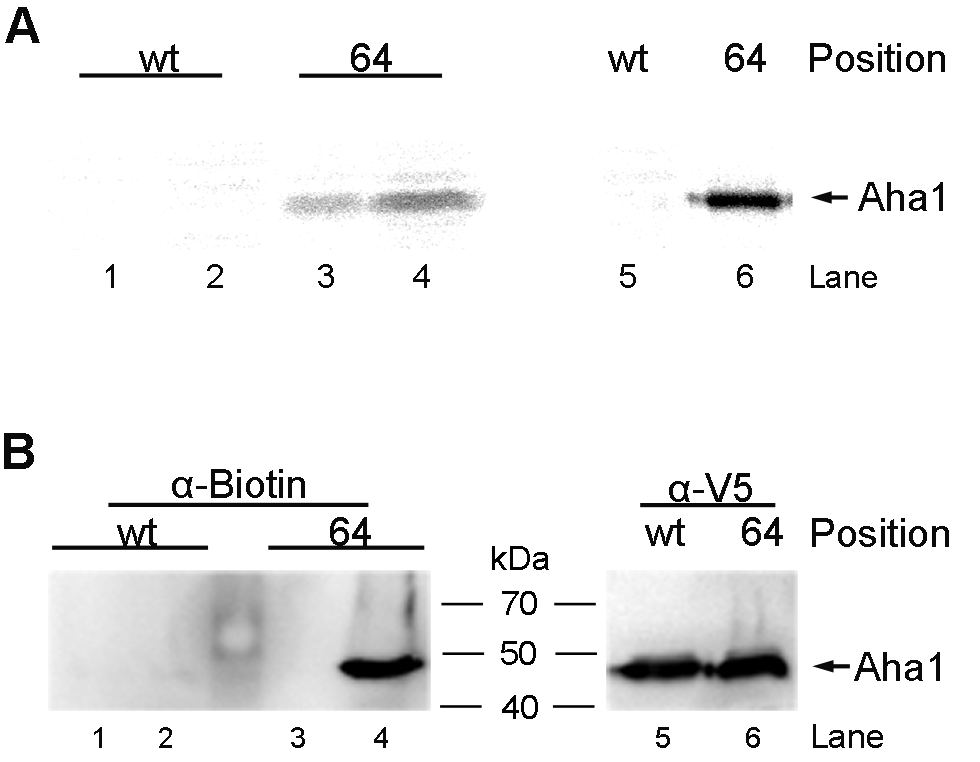

Supplement: Figure S2 — Site-specific conjugation of chemical compounds using the azido-group. A. Chemoselective ligation of the triarylphosphine fluorescent dye using the Staudinger ligation reaction. Aha1 I64X (2YA6,64C1) was expressed in the presence of pAzpa, afterwards cells were disrupted and the ligation reaction was performed using whole cell lysate. Labeled Aha1 protein was immunoprecipitated with anti-V5 antibody. The immunoprecipitate (lane 6) and 50 µg (lane 3) or 100 µg (lane 4) of lysate protein were separated by the SDS-PAGE. Fluorescence was read out by using a fluorescence scanner. Wild-type Aha1 (2YA6C1) was subjected to the same labeling reaction and was used as a negative control (50 µg protein lysate = lane 1; 100 µg protein lysate = lane 2; immunoprecipitated eluate = lane 5). B. Chemoselective conjugation of a biotin molecule using the azide-alkyne cycloaddition (Click Chemistry). Aha1 I64X (2YA6,64C1) was expressed in the presence of pAzpa, afterwards cells were disrupted and Aha1 proteins were immunoprecipitated with the anti-V5 antibody. The eluate was then used for the biotin labeling procedure. Samples before (lane 3) and after (lane 4) labeling were analyzed by Western blot using streptavidin-HRP, showing that biotin was successfully linked to Aha1 at position 64. Labeled Aha1 proteins were also detected with the anti-V5 antibody (lane 6). The wild-type Aha1 (2YA6C1) was used as the negative control before (lane 1) and after (lane 2) labeling; lane 5 represents the same sample as lane 2 but analyzed using anti-V5 antibodies. (TIF) [file pone.0089436.s002.tif]

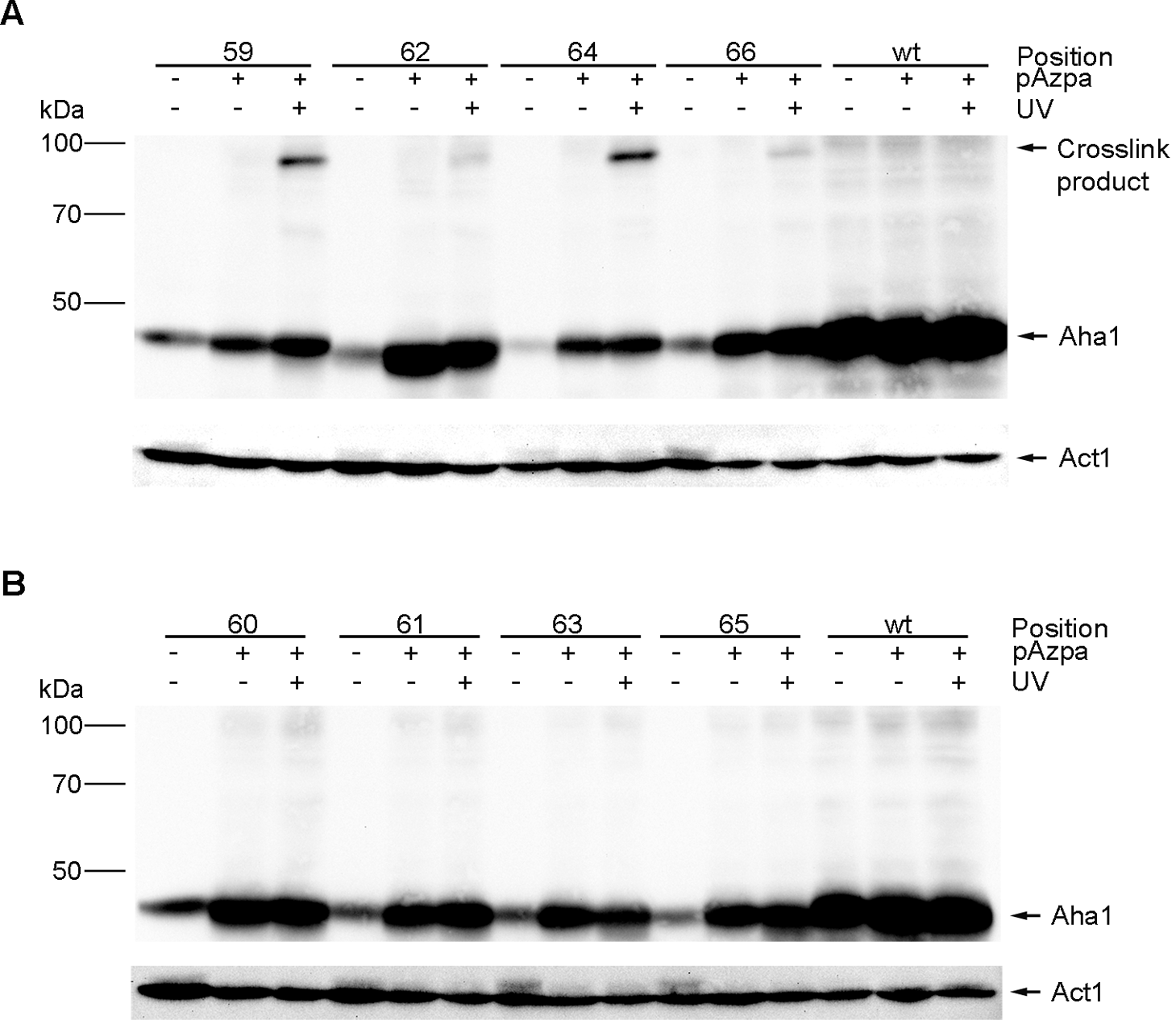

Supplement: Figure S3 — Crosslink product formation depends on position, UV irradiation and the presence of the non-canonical amino acid pAzpa. Wild-type Aha1 (2YA6C1) was used as a negative control and expression of Act1 was used as loading control. Detection of the Aha1 variants was carried out with a monoclonal mouse anti-V5 antibody and the detection of Act1 using a monoclonal mouse anti-Act1 antibody. A. Positions showing crosslink product formation. B. All positions with no crosslink product formation. (TIF) [file pone.0089436.s003.tif]

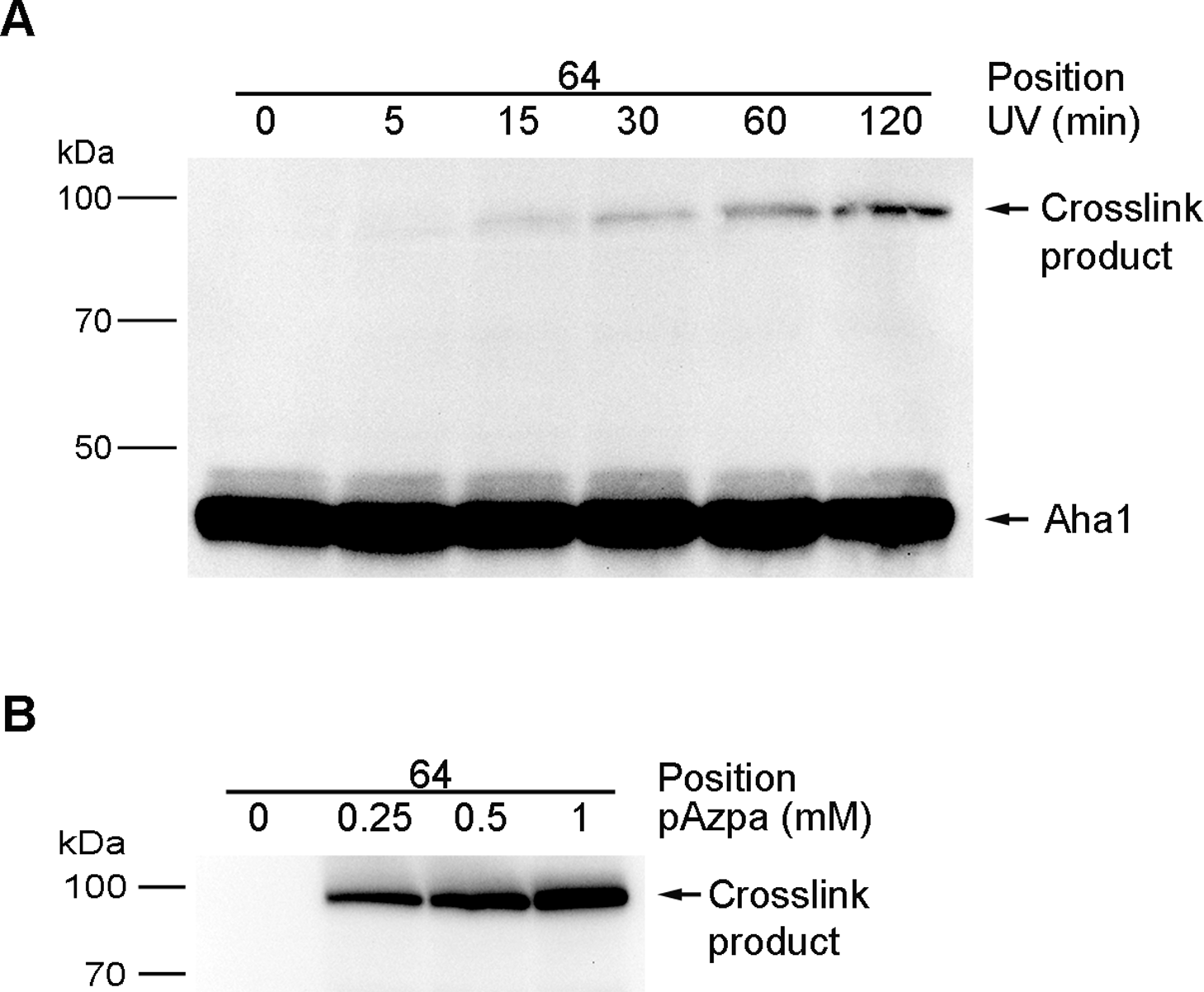

Supplement: Figure S4 — Time of UV irradiation and pAzpa concentration have an influence on crosslink formation. A. Strain 2YA6,64C1 was exposed for different periods of time to UV light. Significant crosslink product formation could be observed after 15 min of irradiation with UV light. Irradiation up to 120 min leads to an increased yield of the formation of the crosslinked product. B. Cells were cultivated in medium with different pAzpa concentrations. The yield of crosslink product formation correlates to the concentration of pAzpa given to the medium. No crosslink product formation was observed without pAzpa. (TIF) [file pone.0089436.s004.tif]

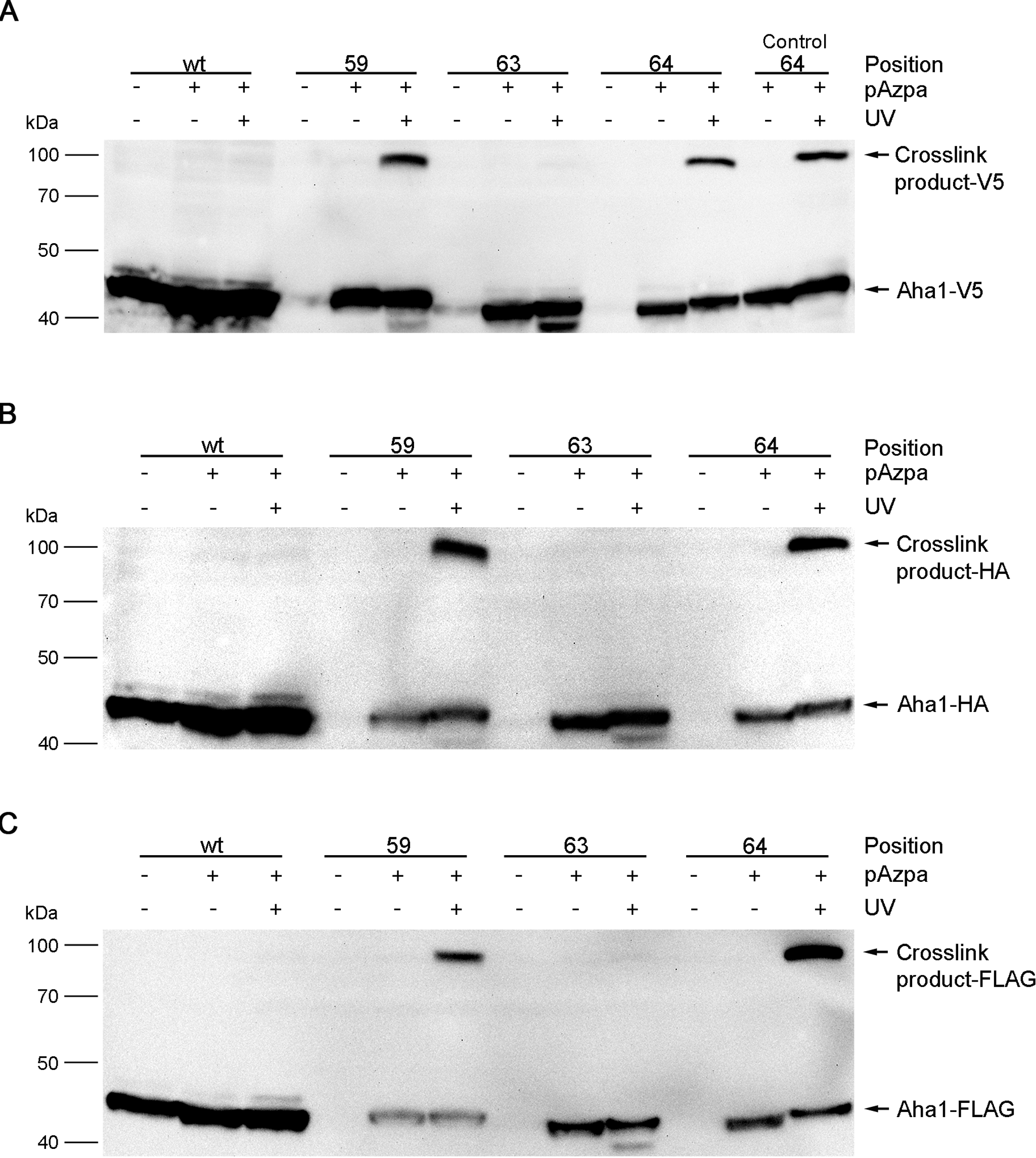

Supplement: Figure S5 — The C-terminal tag has no effect on crosslink product formation. Independent of the C-terminal tag, crosslink product formation could be confirmed with Aha1 variant 59 and 64. A. Different Aha1 variants were tagged with the V5 epitope at the C-terminus. Aha1 I64X (2YA6,64C1) C-terminally tagged with V5 epitope and poly-histidine tag was used as control. B. Aha1 variants C-terminally tagged with the HA epitope detected with anti-HA antibodies. C. Aha1 variants C-terminally tagged with the FLAG epitope and detected with anti-FLAG antibodies. (TIF) [file pone.0089436.s005.tif]

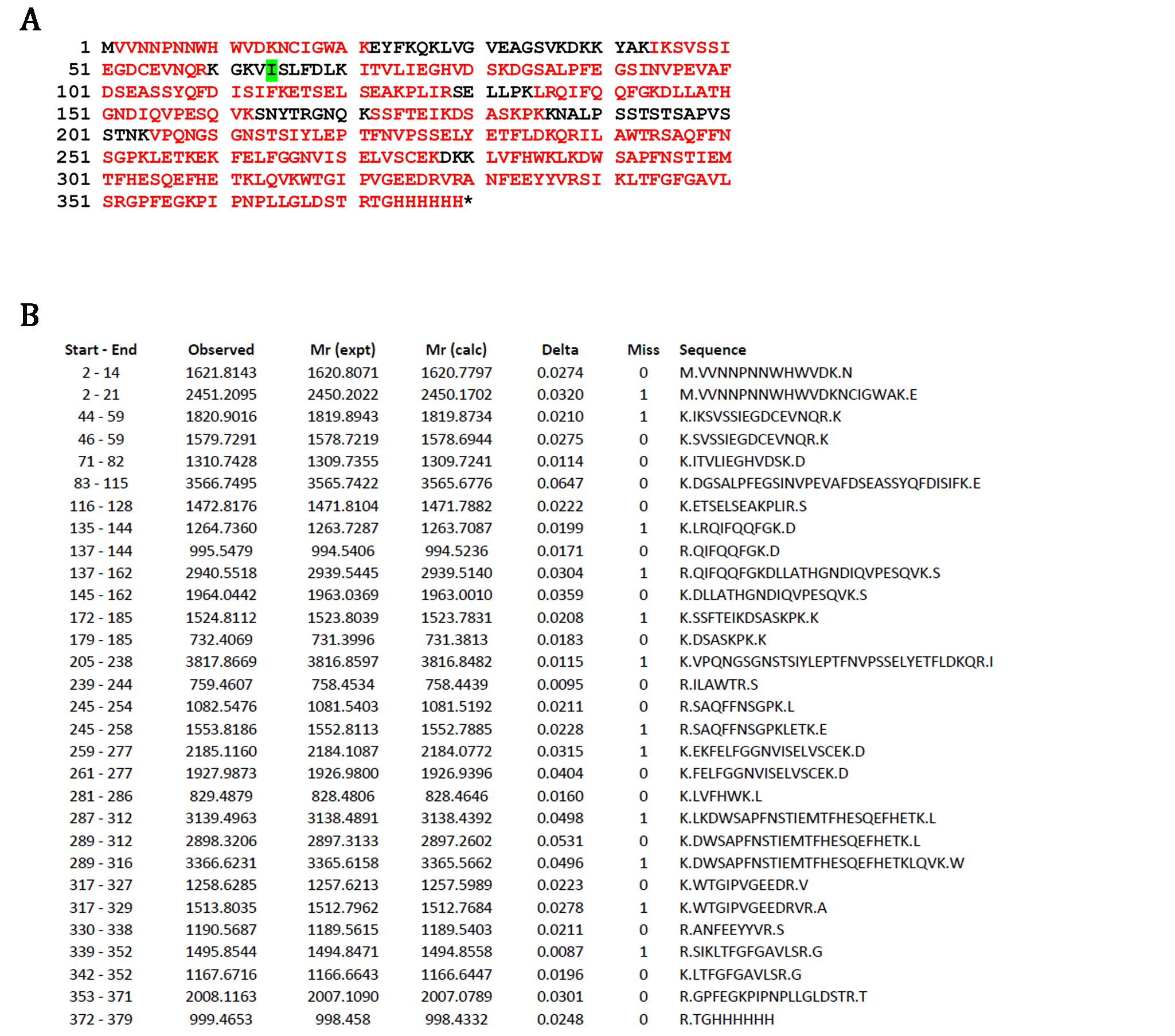

Supplement: Figure S6 — A. Sequence coverage of the Aha1 I64X protein. Protein regions which could be covered by matching peptides are highlighted in red. Position 64 is marked in green. B. Peak table showing all found masses matching to the Aha1 I64X protein sequence. (TIF) [file pone.0089436.s006.tif]

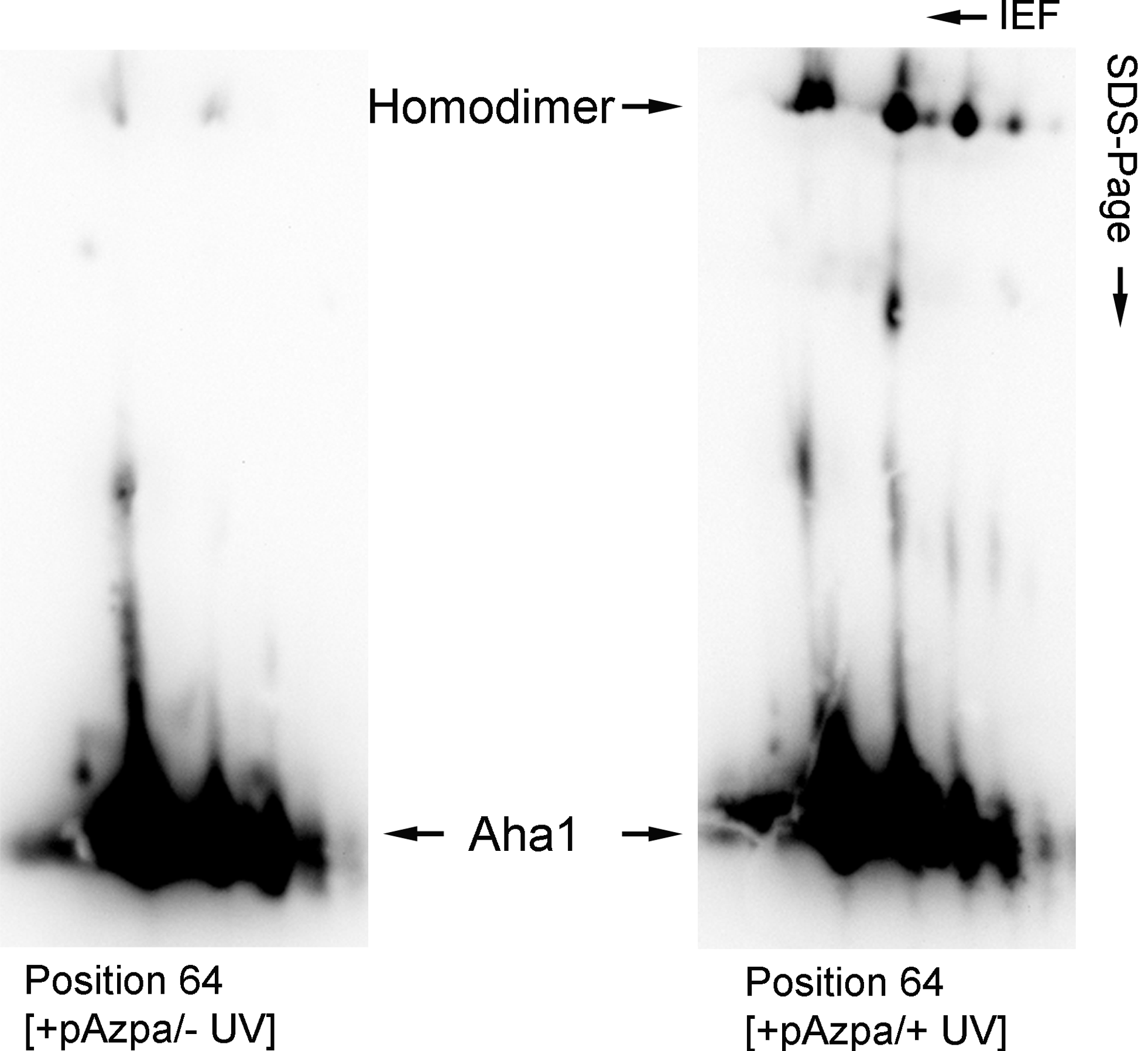

Supplement: Figure S7 — Analysis of the Aha1 homodimer by 2-D electrophoresis. Aha1 I64X (2YA6,64C1) was immunoprecipitated before (left) and after irradiation (right blot) by UV light. Eluates were separated by 2-D electrophoresis and analyzed by Western blot with monoclonal mouse anti-V5 antibody. The crosslink product migrates to the same isoelectric points as the Aha1 monomer. (TIF) [file pone.0089436.s007.tif]

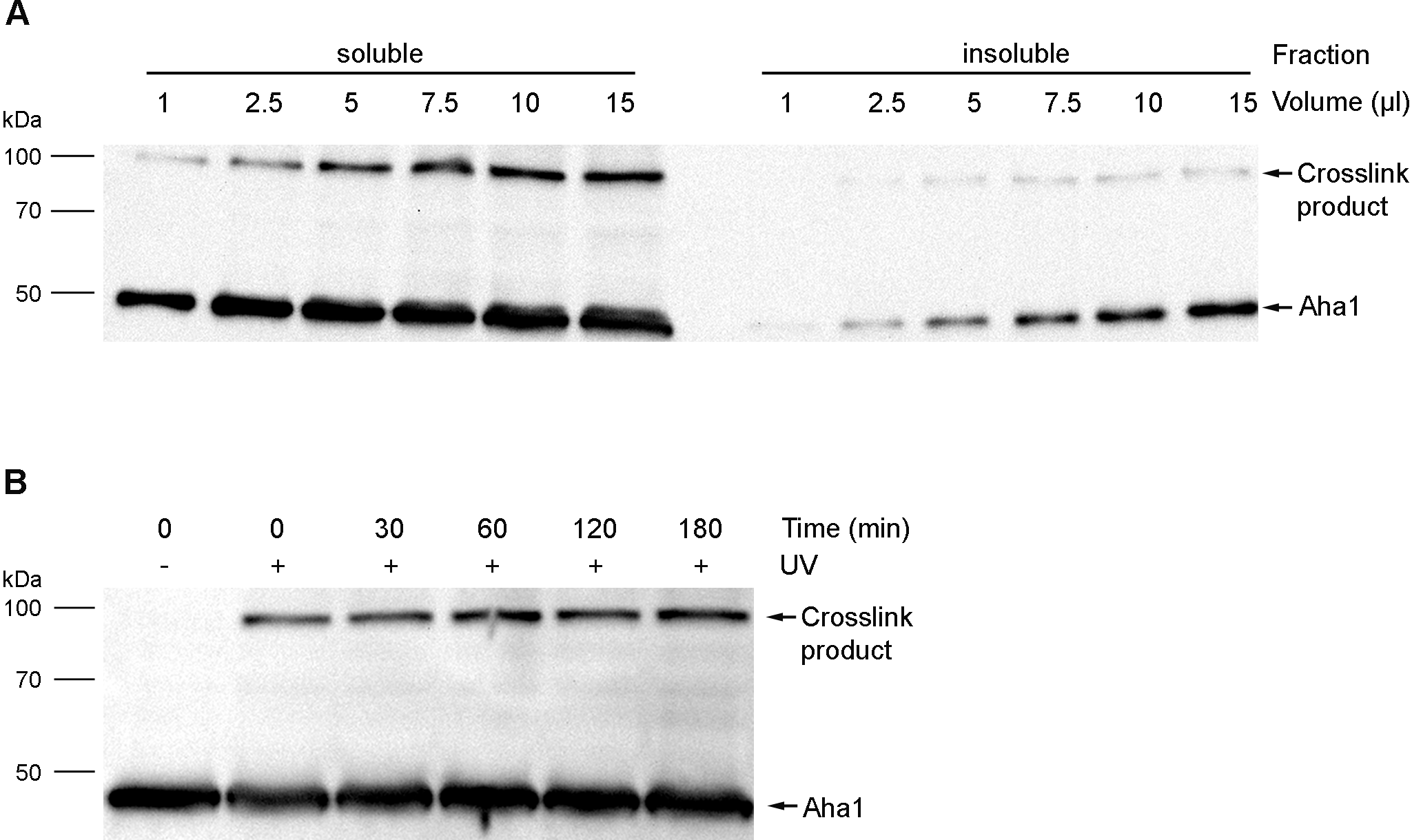

Supplement: Figure S8 — Crosslink products are found in the soluble fraction and are not formed co-translationally at poly-ribosomes. A. Separation of soluble from insoluble proteins was performed to show that the crosslink product is not formed due to the aggregation of misfolded Aha1 proteins within the cell. Aha1 I64X (2YA6,64C1) was expressed, crosslink products were formed by irradiation with UV light and soluble proteins were separated from the insoluble proteins by a 100,000× g centrifugation. Different volumes of each fraction were analyzed by Western Blot using mouse monoclonal anti-V5 antibody. Crosslink product as well as monomer was mainly found in the soluble fraction. B. Cycloheximide-chase assay were performed to demonstrate that crosslinking does not occur co-translationally. At different time points following addition of cycloheximide cells were exposed to UV light. Crosslink products could be formed to each time point after inhibition of the translation in comparable yields. (TIF) [file pone.0089436.s008.tif]
