# Supplementary material for: An In Vivo Photo-Cross-Linking Approach Reveals a Homodimerization Domain of Aha1 in S. cerevisiae
Source: PLoS One. 2014 Mar 10;9(3):e89436. doi: 10.1371/journal.pone.0089436 (PMC3948627; doi:10.1371/journal.pone.0089436)
Supplement: Table S1 — Primer sequences for cloning and mutagenesis of AHA1. (DOCX) [file pone.0089436.s009.docx]

Table S1 Primer sequences

| **Primer** | **5‘ 🡪 3‘ sequence** | **Use** |
| --- | --- | --- |
| AHA1_BamHI | CGGGATCCATGGTCGTGAATAACCCAAA | Amplification of AHA1 |
| AHA1_XbaI | GCTCTAGATAATACGGCACCAAAGCCGAATG | Amplification of AHA1 |
| Aha1_P59_Arg | TGTGAAGTTAATCAGTAGAAGGGGAAGGTTATA | Mutagenesis of codon 59  Mutagenesis of codon 59 |
| revAha1_P59_Arg | ATCACCTTCAATGGACGAAACAGACTTGAT |  |
| Aha1_P60_Lys | GAAGTTAATCAGCGTTAGGGGAAGGTTATATCT | Mutagenesis of codon 60  Mutagenesis of codon 60 |
| revAha1_P60_Lys | ACAATCACCTTCAATGGACGAAACAGACTT |  |
| Aha1_P61_Gly | GTTAATCAGCGTAAGTAGAAGGTTATATCTTTG | Mutagenesis of codon 61  Mutagenesis of codon 61 |
| revAha1_P61_Gly | TTCACAATCACCTTCAATGGACGAAACAGA |  |
| Aha1_P62_Lys | AATCAGCGTAAGGGGTAGGTTATATCTTTGTTT | Mutagenesis of codon 62  Mutagenesis of codon 62 |
| revAha1_P62_Lys | AACTTCACAATCACCTTCAATGGACGAAAC |  |
| Aha1_P63_Val | CAGCGTAAGGGGAAGTAGATATCTTTGTTTGAT | Mutagenesis of codon 63  Mutagenesis of codon 63 |
| revAha1_P63_Val | ATTAACTTCACAATCACCTTCAATGGACGA |  |
| Aha1_P64_Ile | CGTAAGGGGAAGGTTTAGTCTTTGTTTGATTTG | Mutagenesis of codon 64  Mutagenesis of codon 64 |
| revAha1_P64_Ile | CTGATTAACTTCACAATCACCTTCAATGGA |  |
| Aha1_P65_Ser | AAGGGGAAGGTTATATAGTTGTTTGATTTGAAA | Mutagenesis of codon 65  Mutagenesis of codon 65 |
| revAha1_P65_Ser | ACGCTGATTAACTTCACAATCACCTTCAAT |  |
| Aha1_P66_Leu | GGGAAGGTTATATCTTAGTTTGATTTGAAAATC | Mutagenesis of codon 66  Mutagenesis of codon 66 |
| revAha1_P66_Leu | CTTACGCTGATTAACTTCACAATCACCTTC |  |
| S2_AHA1 | GTAAATATTTACGCATACTTTTATTGAAACATGAGAACAATATATCTTAATCGATGAATTCGAGCTCG | PCR-based tagging |
| S3_AHA1 | CTACGTGCGTTCTATTAAATTGACATTCGGCTTTGGTGCCGTATTACGTACGCTGCAGGTCGAC | PCR-based tagging |
