# Supplementary material for: An In Vivo Photo-Cross-Linking Approach Reveals a Homodimerization Domain of Aha1 in S. cerevisiae
Source: PLoS One. 2014 Mar 10;9(3):e89436. doi: 10.1371/journal.pone.0089436 (PMC3948627; doi:10.1371/journal.pone.0089436)
Supplement: Table S2 — S. cerevisiae strains generated in this study. (DOCX) [file pone.0089436.s010.docx]

Table S2 *S. cerevisiae* strains generated in this study

| Name | Based on | Plasmids transfected | Cassette | Reference |
| --- | --- | --- | --- | --- |
| 2Y | YPH501 |  |  | Agilent Technologies |
| 2YA0C1 | YPH501 | pYES2/CT  pAR3/tRNA_CUA_ |  | this work |
| 2YA6C1 | YPH501 | pYES2/CT-AHA1  pAR3/tRNA_CUA_ |  | this work |
| 2YA6,59C1 | YPH501 | pYES2/CT-AHA1(R59TAG)  pAR3/tRNA_CUA_ |  | this work |
| 2YA6,60C1 | YPH501 | pYES2/CT-AHA1(K60TAG)  pAR3/tRNA_CUA_ |  | this work |
| 2YA6,61C1 | YPH501 | pYES2/CT-AHA1(G61TAG)  pAR3/tRNA_CUA_ |  | this work |
| 2YA6,62C1 | YPH501 | pYES2/CT-AHA1(K62TAG)  pAR3/tRNA_CUA_ |  | this work |
| 2YA6,63C1 | YPH501 | pYES2/CT-AHA1(V63TAG)  pAR3/tRNA_CUA_ |  | this work |
| 2YA6,64C1 | YPH501 | pYES2/CT-AHA1(I64TAG)  pAR3/tRNA_CUA_ |  | this work |
| 2YA6,65C1 | YPH501 | pYES2/CT-AHA1(S65TAG)  pAR3/tRNA_CUA_ |  | this work |
| 2YA6,66C1 | YPH501 | pYES2/CT-AHA1(L66TAG)  pAR3/tRNA_CUA_ |  | this work |
| 2YA6C2 | YPH501 | pYES2/CT-AHA1  pBR2/tRNA_CUA_ |  | this work |
| 2YA6,63C2 | YPH501 | pYES2/CT-AHA1(V63TAG)  pBR2/tRNA_CUA_ |  | this work |
| 2YA6,64C2 | YPH501 | pYES2/CT-AHA1(I64TAG)  pBR2/tRNA_CUA_ |  | this work |
| 2YA21C1 | YPH501 | pYES2/CT(V5)-AHA1  pAR3/tRNA_CUA_ |  | this work |
| 2YA21,59C1 | YPH501 | pYES2/CT(V5)-AHA1(R59TAG)  pAR3/tRNA_CUA_ |  | this work |
| 2YA21,63C1 | YPH501 | pYES2/CT(V5)-AHA1(V63TAG)  pAR3/tRNA_CUA_ |  | this work |
| 2YA21,64C1 | YPH501 | pYES2/CT(V5)-AHA1(I64TAG)  pAR3/tRNA_CUA_ |  | this work |
| 2YA31C1 | YPH501 | pYES2/CT(HA)-AHA1  pAR3/tRNA_CUA_ |  | this work |
| 2YA31,59C1 | YPH501 | pYES2/CT(HA)-AHA1(R59TAG)  pAR3/tRNA_CUA_ |  | this work |
| 2YA31,63C1 | YPH501 | pYES2/CT(HA)-AHA1(V63TAG)  pAR3/tRNA_CUA_ |  | this work |
| 2YA31,64C1 | YPH501 | pYES2/CT(HA)-AHA1(I64TAG)  pAR3/tRNA_CUA_ |  | this work |
| 2YA41C1 | YPH501 | pYES2/CT(FLAG)-AHA1  pAR3/tRNA_CUA_ |  | this work |
| 2YA41,59C1 | YPH501 | pYES2/CT(FLAG)-AHA1(R59TAG)  pAR3/tRNA_CUA_ |  | this work |
| 2YA41,63C1 | YPH501 | pYES2/CT(FLAG)-AHA1(V63TAG)  pAR3/tRNA_CUA_ |  | this work |
| 2YA41,64C1 | YPH501 | pYES2/CT(FLAG)-AHA1(I64TAG)  pAR3/tRNA_CUA_ |  | this work |
| 2Y257 | YPH501 |  | pYM45 | this work |
| 2Y257A6C1 | YPH501 | pYES2/CT-AHA1  pAR3/tRNA_CUA_ | pYM45 | this work |
| 2Y257A6,59C1 | YPH501 | pYES2/CT-AHA1(R59TAG)  pAR3/tRNA_CUA_ | pYM45 | this work |
