# Supplementary material for: An In Vivo Photo-Cross-Linking Approach Reveals a Homodimerization Domain of Aha1 in S. cerevisiae
Source: PLoS One. 2014 Mar 10;9(3):e89436. doi: 10.1371/journal.pone.0089436 (PMC3948627; doi:10.1371/journal.pone.0089436)
Supplement: Table S3 — Plasmids used for this work. (DOCX) [file pone.0089436.s011.docx]

Table S3 Plasmids

| Plasmid | Abbreviation | Reference |
| --- | --- | --- |
| pYES2/CT | A0 | Invitrogen |
| pYES2/CT-AHA1 | A6 | this work |
| pYES2/CT-AHA1(R59TAG) | A6,59 | this work |
| pYES2/CT-AHA1(K60TAG) | A6,60 | this work |
| pYES2/CT-AHA1(G61TAG) | A6,61 | this work |
| pYES2/CT-AHA1(K62TAG) | A6,62 | this work |
| pYES2/CT-AHA1(V63TAG) | A6,63 | this work |
| pYES2/CT-AHA1(I64TAG) | A6,64 | this work |
| pYES2/CT-AHA1(S65TAG) | A6,65 | this work |
| pYES2/CT-AHA1(L66TAG) | A6,66 | this work |
| pYES2/CT(V5) | A20 | this work |
| pYES2/CT(V5)-AHA1 | A21 | this work |
| pYES2/CT(V5)-AHA1(R59TAG) | A21,59 | this work |
| pYES2/CT(V5)-AHA1(V63TAG) | A21,63 | this work |
| pYES2/CT(V5)-AHA1(I64TAG) | A21,64 | this work |
| pYES2/CT(HA) | A30 | this work |
| pYES2/CT(HA)-AHA1 | A31 | this work |
| pYES2/CT(HA)-AHA1(R59TAG) | A31,59 | this work |
| pYES2/CT(HA)-AHA1(V63TAG) | A31,63 | this work |
| pYES2/CT(HA)-AHA1(I64TAG) | A31,64 | this work |
| pYES2/CT(FLAG) | A40 | this work |
| pYES2/CT(FLAG)-AHA1 | A41 | this work |
| pYES2/CT(FLAG)-AHA1(R59TAG) | A41,59 | this work |
| pYES2/CT(FLAG)-AHA1(V63TAG) | A41,63 | this work |
| pYES2/CT(FLAG)-AHA1(I64TAG) | A41,64 | this work |
| pAR3-PGK1+3SUP4-tRNA | C1 | [[54](#_ENREF_54)] |
| pBR2-PGK1+3SUP4-tRNA | C2 | [[54](#_ENREF_54)] |
| pYM45 (1 x HA-Tag) | 257 | [[38](#_ENREF_38)] |
